# Behavioural Sciences Contribution to Suppressing Transmission of Covid-19 in the UK: A Systematic Literature Review

**DOI:** 10.1007/s12529-023-10171-4

**Published:** 2023-04-14

**Authors:** Gill Hubbard, Chantal den Daas, Marie Johnston, Jennifer Dunsmore, Mona Maier, Rob Polson, Diane Dixon

**Affiliations:** 1 Department of Nursing, University of the Highlands and Islands, Inverness, UK; 2 Health Psychology Group, University of Aberdeen Institute of Applied Health Sciences, Aberdeen, Scotland; 3 Centre for Health Science, Older Perth Road, Inverness, IV2 3JH UK; 4 School of Applied Sciences, Edinburgh Napier University, 9 Sighthill Court, EH11 4BN Edinburgh, Scotland

**Keywords:** Covid-19, Behavioural science, Public health, Health psychology, Theoretical domains framework

## Abstract

**Background:**

Governments have relied on their citizens to adhere to a variety of transmission-reducing behaviours (TRBs) to suppress the Covid-19 pandemic. Understanding the psychological and sociodemographic predictors of adherence to TRBs will be heavily influenced by the particular theories used by researchers. This review aims to identify the theories and theoretical constructs used to understand adherence to TRBs during the pandemic within the UK social and legislative context.

**Methods:**

A systematic review identified studies to understand TRBs of adults in the UK during the pandemic. Identified theoretical constructs were coded to the Theoretical Domains Framework. Data are presented as a narrative summary.

**Results:**

Thirty-five studies (*n* = 211,209) investigated 123 TRBs, applied 13 theoretical frameworks and reported 50 sociodemographic characteristics and 129 psychological constructs. Most studies used social cognition theories to understand TRBs and employed cross-sectional designs. Risk of sampling bias was high. Relationships between constructs and TRBs varied, but in general, beliefs about the disease (e.g. severity and risk perception) and about TRBs (e.g. behavioural norms) influenced behavioural intentions and self-reported adherence. More studies than not found that older people and females were more adherent.

**Conclusions:**

Behavioural scientists in the UK generated a significant and varied body of work to understand TRBs during the pandemic. However, more use of theories that do not rely on deliberative processes to effect behaviour change and study designs better able to support causal inferences should be used in future to inform public health policy and practice.

**Prospero Registration:**

CRD42021282699.

**Supplementary Information:**

The online version contains supplementary material available at 10.1007/s12529-023-10171-4.

## Introduction

Governments have relied on their citizens to adhere to a variety of transmission-reducing behaviours (TRBs) to suppress Covid-19 transmission. TRBs such as physical distancing, face covering and hand hygiene are critical for suppressing the spread of infectious diseases [1, 2], and governments either mandated or recommended these behaviours at different times over the course of the Covid-19 pandemic. Understanding the sociodemographic and psychological determinants of adherence was key to public health’s efforts to contain the pandemic. Thus, behavioural science theory and empirical evidence for understanding human behaviour and behaviour change had an important role in the worldwide efforts to stem the spread of Covid-19. Infectious disease remains one of the world’s greatest threats to human and animal life, the environment, local communities and economies [3]. Therefore, there is utility in understanding the response of behavioural science to the Covid-19 pandemic with a view to being better prepared to deliver that science to support public health agencies. The following are critical questions: Which theories did behavioural scientists apply and not apply to understand TRBs? What research methods were used? How was the general population stratified? Was the evidence base generated during the pandemic sufficient and available for rapid application by public health colleagues? Answers to these questions will enable the discipline to reflect on its response to the pandemic and to plan for its response to subsequent waves of the current pandemic and the emergence of novel pandemics in the future.

Theory and empiricism are important for understanding behaviour. In the context of understanding behaviour and behaviour change, theory represents knowledge about factors that mediate and moderate behaviour as well as the hypotheses about what human behaviour is and what influences behaviour [4]. A recent review of the application of behaviour change theories within an infectious disease and emergency response context identified the three most commonly cited theories, specifically, the Health Belief Model (HBM), the Protection Motivation Theory (PMT), and the Theory of Planned Behaviour (TPB) [5]. These models/theories emphasise the influence of cognitive processes on behaviour. The HBM has four cognitive constructs: (i) *perceived susceptibility* which refers to perception of risk or vulnerability to a health threat; (ii) *perceived severity* which refers to perception of the seriousness of the health threat; (iii) *perceived benefits* which refers to perceived efficacy of a behaviour to prevent or reduce the threat of illness; and (iv) *perceived barriers* which refers to perception of the negative consequences associated with the behaviour to prevent/reduce the threat of illness [6]. According to PMT, behaviours are influenced by two cognitive processes which are *threat appraisal* and *coping appraisal* [7]. Factors comprising the threat-appraisal process are *perceived severity* of the health threat and *perceived vulnerability* to the health threat; factors comprising the coping-appraisal include *response efficacy*, which is the belief that a behaviour will work to reduce the threat, and *self-efficacy*, which is the perceived ability of being able to actually do the behaviour to ward off the threat [7, 8]. According to the TPB, human behaviour is guided by three beliefs: (i) beliefs about the likely consequences of the behaviour (*behavioural beliefs*); (ii) beliefs about the normative expectations of others (*normative beliefs*); and (iii) beliefs about the presence of factors that may facilitate or impede performance of the behaviour (*control beliefs or self-efficacy*) [9]. There are, however, at least 83 theories of human behaviour and behaviour change with around 1659 overlapping constructs [10] that, as illustrated by the HBM, PMT, and TPB, share similar, if not identical, constructs. The Theoretical Domains Framework (TDF) was produced in order to make these behavioural theories and constructs more accessible by grouping key constructs under broad theoretical domains. The TDF synthesises 128 theoretical constructs from 33 behavioural theories and comprises 14 theoretical domains covering 84 theoretical constructs [11]. It provides a theoretical framework through which to view the cognitive, affective, social and environmental influences on behaviour.

Variation in behaviour has implications when tailoring public health policies, and so it is imperative to understand factors that can explain any behavioural variation. An example of the importance of the gathering of country-specific empirical evidence for understanding behaviour during the Covid-19 pandemic is demonstrated in a study reporting differences in behavioural adherence by geographical region [12]. This review focusses on research conducted in the UK, which represents a specific context that experienced higher Covid-19-related deaths in the first wave of the pandemic relative to other European countries [13]. The objectives of the study were:

1. To list the authors and describe the aims, design, sample and date when data were collected of studies about TRBs during the Covid-19 pandemic in the UK
2. To assess study quality
3. To describe behavioural theoretical frameworks informing studies
4. To describe psychological and sociodemographic variables investigated and identify which were associated with transmission-reducing behaviour and behaviour intention
5. To summarise current knowledge and understanding of behaviours and identify gaps in evidence

## Method

### Design

The study design was a systematic review of the literature registered on Prospero (CRD42021282699). The research team included six behavioural scientists from the Covid-19 Health Adherence Research in Scotland (CHARIS) project (CD, DD, GH, MJ, MM, JD) and an information specialist (RP).

### Eligibility Criteria

Eligible studies were about the TBRs of the UK general public during the Covid-19 pandemic, published before 3 September 2021, and included people of any age, gender and ethnicity. Studies about sub-groups of the population, for example, older people, were included if the setting (context for behaviour) was for the general population. Studies about behaviours of clinical populations and research conducted in specific settings such as students in schools and people in hospitals and care homes were excluded. Studies had to report levels of adherence to TRBs/intention and/or psychological and sociodemographic predictors of TRB/intention. Studies about vaccination were excluded because vaccination is an invasive procedure (i.e. a medical procedure that invades (enters) the body, usually by cutting or puncturing the skin or by inserting instruments into the body) whereas the other behaviours are non-invasive. Studies about shielding were excluded because it is a behaviour that only applied to a section of the general population. Epidemiological modelling studies, protocols, service evaluations and qualitative studies were excluded. Studies that were conducted in several countries were included if UK data were reported separately. Articles had to be available in the English language (see Supplementary File Table S1).

### Information Sources and Search

The search was conducted on 3 September 2021, and reported in line with the PRISMA statement [14]. Searches were carried out by an information specialist (RP) in OVID (Embase, Medline, PsychInfo), SCOPUS, Ebsco CINAHL and Psyarxiv (all searches in Supplementary File 2). Search results from the electronic databases were exported to an Excel file and Endnote referencing management tool. Duplications were identified during the screening process and removed.

### Article Selection

Two researchers (GH, CD) screened titles and abstracts of the records retrieved. All papers included by both reviewers were brought forward for full-text review. For papers that were only identified for inclusion by one of the reviewers, a third reviewer (DD) decided if it was to be brought forward for full-text review. Full texts of the remaining included articles were then obtained, independently reviewed and checked for eligibility by two researchers (GH, DD) who resolved any disagreement by consensus (percentage agreement was 75%).

### Data Extraction

Data extraction forms were created using Microsoft Excel. Data extracted were as follows: study date, title, aim, design, location, sample, period of data collection, TRBs investigated, theory applied, psychosocial and sociodemographic variables included in the analysis to determine associations with behaviour and theoretical frameworks/models informing the study. Three researchers (GH, MM, JD) independently extracted data, and any disagreements were resolved via consensus. If the study included univariate and multivariate regression analyses of associations between psychosocial/sociodemographic variables and TRBs, then only the adjusted results of the final regression model were extracted. After data extraction, two researchers (MJ, GH) independently categorised psychological variables to one or more of the 14 theoretical domains within the theoretical domains framework [11]. Disagreements were less than 10% and were resolved by consensus discussion between MJ and GH. For example, if a study assessed the influence of risk perceptions and self-efficacy on TRBs, then the study was listed under domain six, ‘beliefs about consequences’, and domain four, ‘beliefs about capabilities’. One researcher (GH) collated behaviours into one of two types as identified in previous pandemics: ‘avoidant behaviours’, which were defined as interpersonal behaviours concerned with proximity to other people, or ‘preventive behaviours’, which were defined as personal behaviours concerned with proximity to the virus [10].

### Quality Assessment

The Appraisal tool for Cross-Sectional Studies (AXIS) [15] was used to assess study quality by three researchers independently (GH, MM, JD). The tool assesses the introduction, methods, results and discussion with 19 items. All articles scored highly on all items but sampling. Hence, we report the 10 items relating to sampling.

### Data Summary and Synthesis

High levels of heterogeneity precluded statistical pooling. Therefore, the Synthesis without meta-analysis in systematic reviews: reporting guideline [16] guided the narrative summary.

## Results

### Study Selection

**Fig. 1:**
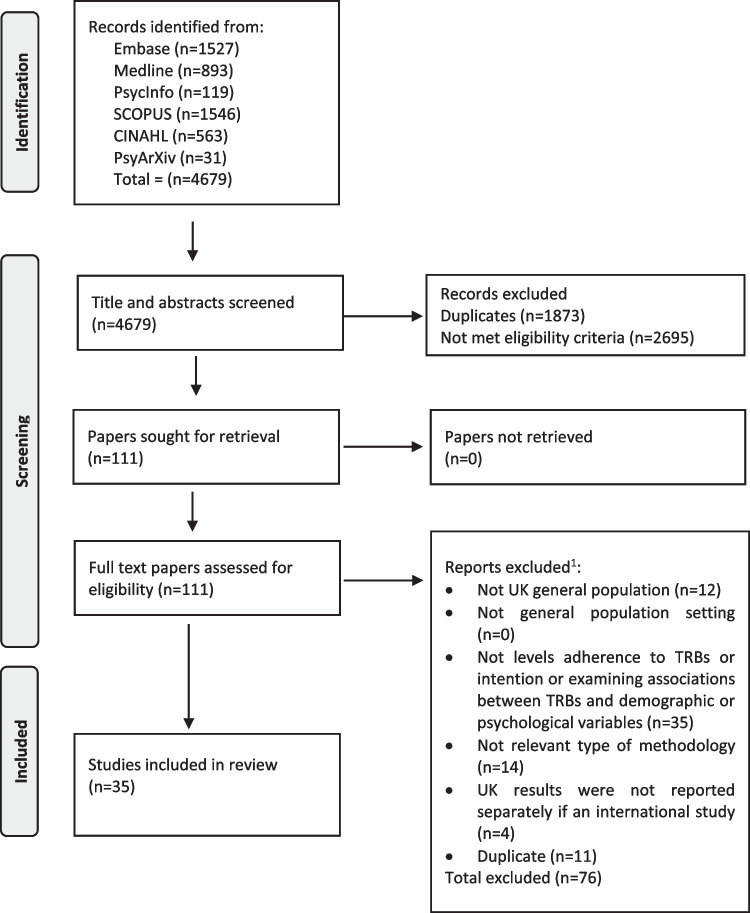
PRISMA flowchart. ^1^The first reason for exclusion is recorded even if the article did not meet other criteria

The search identified 4679 articles; 1873 duplicates were removed; 2695 articles did not meet the eligibility criteria. After the screening of titles and abstracts, 111 papers were brought forward for full-text assessment for eligibility. After full-text screening, 35 papers were brought forward for data extraction (Fig. 1).

### Quality

The general finding from the quality appraisal is that risk of sampling bias was high (Supplementary File 1 Table S2). Twenty-four (68%) studies used existing participant panels of market research companies to recruit respondents using applied non-probability quota sampling. Few reported the response rate, non-responders, representativeness or responders not included in the analysis.

### Design of Studies

**Fig. 2:**
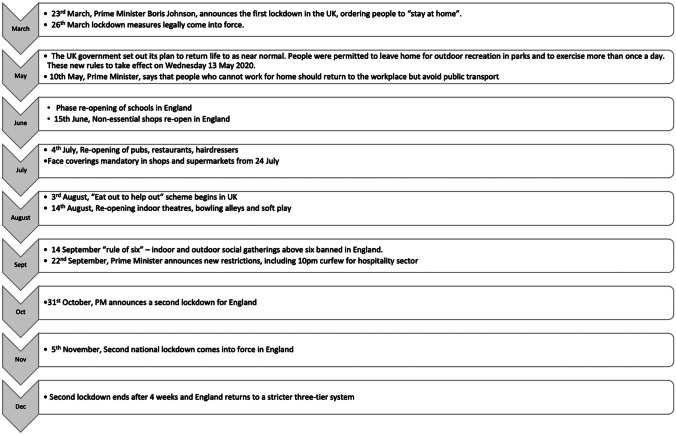
Main changes in lockdown restrictions, March 2020–December 2020. Data from Institute for Government: https://www.instituteforgovernment.org.uk/charts/uk-government-coronavirus-lockdowns

Thirty-five articles reported the levels of adherence to TRBs/intentions and/or sociodemographic and/or psychological constructs associated with adherence (Table 1). The total sample size of all studies was 211,209 (range, 130–53,880). Fifty-four percent (*n* = 19) were cross-sectional studies, and 17% were repeated cross-sectional studies (*n* = 6). Twenty-seven (77%) were UK studies including people from one or more of the devolved nations. The majority (77%) were conducted during the first wave of the pandemic when the UK was in lockdown (23 March 2020 to 30 May 2020) [17]. Figure 2 highlights key milestones in England (chosen because it is the UK nation with the largest population) over this period and shows that studies took place during or in between lockdowns.

**Table 1:** Included studies

**Author**	**Date data was collected**	**Wave**^b^	**Design**	**Main aim**	**Sample**
**Armitage et al. **[54]	30 April 2020	1st	Cross-sectional	To assess levels of public adherence to government instructions to reduce transmission of SARS-CoV-2, but more importantly to gauge why people were or were not adhering to instructions	UK
**Atchison et al.**^d^ [55]	17–18 March 2020	1st	Cross-sectional	To examine risk perceptions and behavioural responses of the UK adult population during the early phase of the Covid-19 epidemic in the UK	UK (4 nations)
**Bacon and Corr** [56]	18–19 March 2020	1st	Cross-sectional	To examine the role of personality factors in concerns about coronavirus, personal safety and the intention to self-isolate through the lens of the reinforcement sensitivity theory (RST) of personality	UK
**Bowman et al.**^d^ [57]	17–18 March 2020	1st	Cross-sectional	To compare psychobehavioural responses in Hong Kong and the UK during the early phase of the Covid-19 pandemic	UK^a^
**Dixon** [18]	3 June and 15 July 2020	Between 1^st^ and 2nd	Repeated cross-sectional	To examine the ability of four models of behaviour, namely, Protection Motivation Theory (PMT), the Common Sense Self-Regulation Model (CS-SRM) and Social Cognitive Theory and the Reasoned Action Approach (SCT and RAA) to understand adherence to transmission-reducing behaviours	Scotland
**Dowthwaite et al.** [58]	11–21 December 2020	2nd	Cross-sectional	To investigate adoption of and attitudes toward the NHS (National Health Service) Covid-19 smartphone app, the digital contact tracing solution in the UK	UK (4 nations)
**Eraso and Hills**^**b**^ [24]	1–31 May 2020	1st	Mixed methods—including cross-sectional survey	To investigate non-adherence behaviours to self-isolation and quarantine measures, their potential predictors and people’s accounts of their experiences	London
**Eraso and Hills**^a^ [21]	1–31 May 2020	1st	Mixed methods—including cross-sectional survey	To investigate non-adherence to a cluster of social distancing rules (keeping 2 m distancing, meeting family and friends and going out for non-essential reasons)	London
**Fujii et al. **[59]	15–23 April 2020	1st	Cross-sectional	To assess whether self-reported perceptions of Covid-19 and personal characteristics are associated with protective behaviours amongst general adults and to compare patterns in six different countries	UK
**Galasso et al.** [60]	16–20 March and 15–20 April 2020	1st	Repeated cross-sectional	To investigate gender differences in Covid-19 − related beliefs and behaviours	UK^a^
**Hills and Eraso**^c^ [22]	1–31 May 2020	1st	Cross-sectional	To investigate: (1) What are the demographic, housing, health, political, psychological and social factors associated with non-adherence of all SD rules by North London residents? (2) What are the demographic, housing, health, political, psychological and social factors associated with intentional non-adherence of SD rules by North London residents?	London
**Jain et al.** [61]	17 July–10 Sept 2020	Between 1^st^ &2^nd^ wave	Retrospective cohort	To identify the proportion of symptomatic staff members attending workplaces after symptom onset or testing, and associated factors	London
**Jorgensen et al.** [28]	19 March–16 May 2020	1st	Repeated cross-sectional	To investigate who is most likely to comply with advice from health authorities	UK (4 nations)^a^
**Keyworth et al.** [62]	30th April 2020	1st	Cross-sectional	To evaluate challenges to adhering to government instructions	UK
**Lawson et al.** [63]	10–20 March 2020	1st	Direct observational	To evaluate hand hygiene behaviour of the general public	Northern Ireland
**MacIntyre et al.** [64]	10 July–27 July 2020		Cross-sectional	To determine patterns of mask wearing and other infection prevention behaviours	London^a^
**Maher et al.** [27]	9 March–6 April 2020	1st	Network visualisation	To map the emergence of opposing opinion-based groups and assess their implications for behaviour	UK
**Margraf et al.** [65]	May–June 2020	1st	Cross-sectional	To investigate the perceived usefulness, adherence and predictors of behaviours	UK^a^
**Norman et al.** [19]	April 2020	1st	Cross-sectional	To examine associations between demographics, people’s beliefs and compliance with behaviours	UK
**Perrotta et al.** [66]	13 March–19 April 2020	1st	Cross-sectional	To investigate how different demographic groups of respondents differ in (i) their perception of the threat posed by Covid-19, (ii) their confidence in the preparedness of various organisations to handle the pandemic and (iii) the uptake of preventive and social distancing behaviours	UK^a^
**Schneider et al.** [67]	March 2020–January 2021	1^st^ and 2nd	Repeated cross-sectional	To investigate public risk perception of Covid-19 and its association with health protective behaviours	UK
**Schuz et al.** [20]	April 2020	1st	Cross-sectional	To examine whether relationships between health cognitions based on the Reasoned Action Approach (RAA) and eight Covid-19 protection behaviours vary as a function of participant-level socio- structural factors	UK
**Shiina et al.** [31]	April 17, 2020	1st	Cross-sectional	To examine between-country differences in perception, attitude and precautionary behaviours toward Covid-19	UK^a^
**Smith et al.**^a ^[26]	6–7 May 2020	1st	Cross-sectional	To investigate factors associated with adherence to self-isolation and lockdown measures due to Covid-19	UK
**Smith et al.**^**b**^ [68]	20–22 April 2020	1st	Cross-sectional	To investigate whether people who think they have had Covid-19 are less likely to report engaging with lockdown measures compared with those who think they have not had Covid-19	UK (4 nations)
**Smith et al.**^**c**^ [23]	March 2020–January 2021	1st	Repeated cross sectional	To investigate rates of adherence to the UK’s test, trace and isolate system	UK (4 nations)
**Swami et al.** [69]	April 2020	1st	Cross-sectional	To examine the extent to which both rejection of Covid-19 conspiracy theories/theorists and rational thinking style are related to compliance with mandated requirements to stop the spread of Covid-19	UK
**Woelfert and Kunts **[70]	15 March 2020	1st	Mixed methods: cross-sectional and experiment	To examine the interplay of political trust, social trust and social distancing	UK
**Wright and Fancourt **[71]	1 April–31 August 2020	Between 1^st^ and 2^nd^	Repeated cross-sectional	To determine the extent to which demographic, socio-economic position, personality traits, social and prosocial motivations and the living environment predict compliance changed across the pandemic	UK (4 nations)
**Wright et al.** [25]	1 April–22 June 2020	Between 1^st^ and 2^nd^	Prospective, longitudinal	To determine whether within-person changes in confidence in government, mental wellbeing, social experiences and awareness of Covid-19 were longitudinally related to self-reported compliance levels with guidelines from authorities	UK (4 nations)
**Juanchich et al.** [29]	20 March–30 April	1st	Cross-sectional	To (i) identify the prevalence of Covid-19 conspiracy theories in the UK, (ii) map their socio-psychological predictors and (iii) investigate their association with health safeguarding behaviours	UK
**Krekal et al.** [72]	23 March–10 May 2020	1st	Prospective, longitudinal	To determine whether happier people are more willing to comply with these measures	UK^a^
**Raihani and de-Wit** [73]	12–24 March 2020	1st	Prospective, longitudinal	To explore (i) subjective concern about the health impacts of Covid-19 19 for self, for family and for society; (ii) the factors associated with compliance with several 20 preventive behavioural measures; and (iii) support for policy measures to reduce the spread 21 of the virus	UK^a^
**Lewis et al.** [74]	12 September–22 October 2020	2nd	Cross-sectional	To examine whether capability, opportunity and motivation significantly predicted intentions to self-isolate again amongst Covid-19 contacts	Wales
**Gould et al.** [75]	15 May–20 May 2021	1st	Direct observational	To investigate how (1) context within a venue, (2) environmental design, (3) staffing and social norms and (4) time across an event, affected personal protective behaviours of social distancing, face covering use and hand hygiene	Wales

^a^Study included as part of a multi-country international study

^b^The estimated dates for the first wave are 23 March 2020 to 30 May 2020, and the estimated dates for the second wave are 7 September 2020 to 24 April 2021[17]

^c, d^A study in same the project that reported a different set of results in separate manuscripts

Thirty-two (91%) studies investigated adherence to behaviours to reduce viral transmission (TRBs) and nine studies investigated TRB intentions (including four studies that also measured adherence to TRBs). Below, we use TRB when citing studies that report behaviour only, intentions when citing studies that report intentions only and TRB/intentions when citing studies that report behaviour and/or intentions. Twenty-two (63%) reported associations between sociodemographic constructs and TRBs/intentions, and 24 (68%) between psychological constructs and TRBs/intentions (Supplementary File 1 Table S3).

### Behaviours

One hundred and twenty-three different TRBs were investigated (Table 2). TRBs most frequently investigated during the first wave of the pandemic were two avoidant behaviours that were social gatherings and leaving the house and two preventive behaviours that were hand hygiene and face covering. Nineteen studies (51%) investigated nine behaviours relating to social gatherings (avoidant behaviour) which were avoiding crowds, social events/activities, places where large group of people come together, social gatherings of more than 20 people, restricting visitors, not being in a room with 10 people, not meeting family members you do not live with, not meeting friends you do not live with and not visiting/meeting family or friends you do not live with; sixteen studies (46%) investigated 27 behaviours relating to leaving the house (avoidant behaviour), seventeen (47%) investigated 16 behaviours relating to hand hygiene (preventive behaviour) and 14 (37%) investigated five behaviours relating to face covering (preventive behaviour). One of the least studied behaviours was Covid-19 testing (*n* = 3 studies, 8%), which is probably because most of the studies included in this review were conducted prior to the introduction of ‘test and trace’ in England and its equivalent in other UK nations.

**Table 2:** Behaviours measured

**Author**	**Adhere to government instructions**	**Avoidant behaviours**						**Preventive behaviours**				
		**Physical distancing**^a^	**Contact with other people**^b^	**Social gatherings**^**c**^	**Leaving the house**^d^	**Travelling**^e^	**Self-isolating**^f^	**Face covering**^g^	**Hand hygiene**^h^	**Other personal hygiene behaviours**^i^	**Cleaning**^j^	**Covid-19 testing**^k^
Number of behaviours	–	5	11	9	27	9	2	5	16	10	5	3
Armitage	x											
Atchison			x	x	x	x	x	x	x	x	x	
Bacon							x					
Bowman			x	x	x	x		x	x	x	x	
Dixon		x						x	x			
Dowthwaite							x					x
Eraso^b^							x	x				
Eraso^a^				x	x			x				
Fujii				x				x	x			
Galasso		x	x	x	x			x	x	x		
Hills				x	x							
Jain							x					
Jorgensen		x	x	x		x			x	x	x	
Keyworth	x											
Lawson									x			
MacIntyre		x	x	x	x			x		x		
Maher		x							x			
Margraf	x											
Norman		x		x	x				x			
Perrotta			x	x		x		x	x			
Schneider					x	x		x	x	x		
Schuz		x		x	x			x	x			
Shiina			x	x	x	x		x	x			
Smith^a^				x	x							
Smith^b^				x	x							
Smith^c^							x					x
Swami		x		x	x				x			
Woelfert		x	x	x								
Wright	x											
Wright/Steptoe	x											
Juanchich		x	x	x	x	x	x	x	x	x	x	x
Krekel	x		x	x	x	x	x	x	x	x	x	
Raihani			x	x	x	x			x	x		
Lewis							x					
Gould								x				
**Total**	**6**	**10**	**11**	**19**	**16**	**9**	**9**	**14**	**17**	**9**	**5**	**3**

^a^Physical distancing; keeping safe distance from others; keeping distance from people outside closest family; keeping 1/1.5/2-m distance, 2-m distance inside shop, 2-m distance when outside home

^b^Contact with people with fever or respiratory symptoms, contact with people who had been in affected areas in the UK, avoiding in-person contact with others, avoiding people who cough and sneeze, contact with sick people, avoiding people who have come in contact with infected people, contact with elderly and chronically ill people, handshakes and other personal contact, shaking hands or hugging, hugging or kissing outside close family, shaking someone’s hand

^c^Crowds, avoiding places where large groups of people come together, social gathering of more than 20 people, restricting visitors, in room with 10 people, meeting family you do not live with, meeting friends you do not live with, visiting/meeting family or friends you do not live with, social events/activities

^d^Leaving homes less than once a day; limiting yourself to one session of exercise (e.g. walk, run, cycle) close to home each day; going out to parks and public spaces or for exercise; going out for groceries; shopping for groceries/pharmacy; shopping for other things than groceries/pharmacy; leaving home each week to shop for food; leaving home to go to shops for groceries, toiletries or medicine; leaving home for exercise; leaving home to help someone else; leaving home for a medical purpose excluding going to shops/pharmacy for medicine; staying at home; going out for medication; going out for food shopping, exercise, medical needs or travelling to work; cooking at home; going to healthcare settings; going to work; avoiding school and work; work from home; work events (meetings, conferences); number of times went out; shopping; stockpiling food/medicines; purchasing extra supplies; going to school/letting children go to school; going to wet market

^e^Travel, travelling to infected areas of world, travelling to other countries outside UK regardless of whether infected, avoiding travelling to areas with high infection, travelling to other areas of UK regardless of whether infected, travel within UK, travel abroad, public transport, taxi

^f^Self-isolating, self-isolating in own room

^g^Face covering, face covering when away from home, face covering in public spaces, face covering when in a shop, face covering when travelling on public transport

^h^Washed hands as soon as got home, clean hands, washing hands before eating snacks, washing hands for 20 s, washing hands after sneezing or coughing, washing hands after touching something, washing hands with soap and water, washing hands more often, number of times washed hands, using hand sanitiser, hand hygiene intention (did the person go to wash their hands?), hand drying intention (did the person go to dry their hands), hand drying compliance (what method was used to dry hands), time spent drying hands, practising hand hygiene (washing hands, using sanitisers, avoiding touching face), washing hands or using hand sanitiser

^i^Touching face, covering nose and mouth when coughing/sneezing, putting used tissues in the bin straightaway and washing hands afterwards, coughing into elbow, coughing into sleeve, wearing gloves, wearing other types of PPE (gloves, face shields), touching eyes and mouth

^j^Cleaning/disinfecting, using disinfectant, washing all shopping products with soap, unpacking everything, throwing the packaging and washing hands, putting outside shopping and packages

^k^Test and trace app, antibody test, sharing details of close contact

### Sociodemographic Associations with Behaviour

Fifty sociodemographic independent variables were included in the analyses to determine their association with TRBs/intentions. Twenty (57%) studies investigated gender, 20 (57%) age, 11 (31%) ethnicity, 10 (28%) employment and 10 (28%) education (Table 3). There is no clearly consistent pattern of statistically significant association; out of the 20 studies that investigated gender, 11 (55%) studies found that females were more adherent than males; out of the 20 studies that investigated age, 11 studies (55%) found that older people were more adherent than young people; out of the 11 studies that investigated ethnicity, 9 (82%) studies did not find any differences and 2 (18%) studies found that people of white ethnicity were more adherent; out of the 10 studies that investigated employment, 6 (60%) studies did not find any differences and 3 studies (30%) found that people who were not working/not working full time were more adherent; and out of the 10 studies that investigated education, 6 (60%) studies did not find any difference, 2 (20%) studies found that people with poorer education were more adherent and 2 (20%) studies found that people with greater education (for example, educated to degree level) were more adherent (Supplementary File 1 Table S4).

**Table 3:** Sociodemographic independent variables included in the analysis to determine associations with TRBs/intentions

**Socio-demographic variables**	**Studies**	**Total**
Gender^a^	Armitage, Atchison, Bacon, Dixon, Eraso^a^, Eraso^b^, Fujii, Galasso, Hills, Jain, Lawson, Margraf, Norman, Schuz, Smith^a^, Smith^c^, Woelfert, Wright^a^, Juanchich, Raihani	20
Ethnicity	Armitage, Atchison, Dixon, Dowthwaite, Eraso^a^, Eraso^b^, Hills, Norman, Schuz, Smith^c^, Wright^a^	11
Age	Armitage, Atchison, Bacon, Dixon, Dowthwaite, Eraso^a^, Eraso^b^, Fujii, Hills, Jain, Lawson, Margraf, Norman, Schuz, Smith^a^, Smith^c^, Woelfert, Wright^a^, Juanchich, Raihani	20
Employment	Armitage, Atchison, Dixon, Eraso^a^, Eraso^b^, Hills, Jain, Smith^a^, Smith^c^, Wright^a^	10
Education	Atchison, Eraso^a^, Eraso^b^, Hills, Maher, Smith^a^, Smith^c^, Woelfert, Wright^a^, Juanchich	10
Area deprivation	Dixon, Eraso^a^, Eraso^b^, Hills, Norman, Schuz, Smith^a^, Smith^c^	8
Adults in household	Dixon, Eraso^a^, Eraso^b^, Fujii, Hills, Smith^a^, Smith^c^, Wright^a^	8
Children	Dixon, Margraf, Smith^a^, Smith^c^, Wright^a^	6
Physical health^e^	Dixon, Eraso^a^, Eraso^b^, Hills, Margraf, Smith^a^, Smith^c^, Wright^a^, Raihani	9
Mental health^f^	Bowman, Fujii, Margraf, Wright^a^, Wright^b^	5
Covid-19 status^g^	Bacon, Dixon, Smith^a^, Smith^b^, Smith^c^	5
Location^c^	Atchison, Dixon, Fujii, Smith^a^, Smith^c^, Woelfert, Wright^a^	7
Housing tenure	Atchison, Dixon, Eraso^a^, Eraso^b^, Hills	5
Social status	Bacon, Maher, Margraf, Smith^a^, Smith^c^	5
Finance^b^	Atchison, Fujii, Maher, Woelfert, Wright^a^, Juanchich	6
Marital status	Atchison, Margraf, Smith^a^, Smith^c^	4
English first language	Eraso^b^, Eraso^a^, Hills	3
Religion	Eraso^b^, Eraso^a^, Hills	3
Neighbourhood^d^	Margraf, Smith^a^, Wright^a^	3
Pet ownership	Smith^a^	1
User of public transport	Fujii	1

^a^All studies used the term ‘gender’ except Atchison, Fujii, Norman and Schuz who used the term ‘sex’

^b^Personal income, household income, savings, hardship (*n* = 4)

^c^UK nation, region of country, urbanity/rurality (*n* = 3)

^d^Living environment, number of rooms in dwelling, outside space, neighbourhood social capital, neighbourhood attachment, neighbourhood satisfaction, available neighbourhood space, neighbourhood crowding (*n* = 8)

^e^Medical condition associated with vulnerability to Covid-19, Covid-19 risk group due to older age or medical condition, living with someone who is vulnerable, shielding status, shielding due to pre-existing condition, a family member shielding, home for other reason than shielding, long-term conditions, physical health state, underlying health issue affecting day-to-day life (*n* = 10)

^f^Psychiatric condition, anxiety, depression, mental health state (*n* = 4)

^g^Self or member of household with symptoms for Covid-19, have or had Covid-19, self or someone close tested positive for Covid-19, if self-isolating, if believed had Covid (*n* = 5)

### Behaviour Theoretical Frameworks

Fifteen studies (43%) were explicitly informed by a behavioural theoretical framework/model (Table 4). The most common were Protection Motivation Theory (*n* = 5) and the Theory of Planned Behaviour and its derivative, Reasoned Action Approach (*n* = 5), which focus on the influence of cognitive processes on behaviour.

**Table 4:** Theoretical frameworks/models informing studies

**Theory/no theory**	**Study**
No theory used	Atchison, Bowman, Dowthwaite, Fujii, Galasso, Jain, Juanchich, Keyworth, Lawson, MacIntyre, Maher, Perotta, Raihani, Schneider, Shiina, Smith^b^, Smith^c^, Woelfert, Wright^a^, Wright^b^
COM-B	Armitage, Lewis
Reinforcement sensitivity theory of personality	Bacon
Common sense self-regulation model	Dixon
Reasoned Action Approach	Dixon, Norman, Schuz
Protection Motivation Theory	Dixon, Hills, Jorgensen, Margraf, Smith^a^
Social Cognitive Theory	Dixon
Social Ecological Theory	Eraso^a^, Eraso^b^, Hills
Theory of Planned Behaviour	Eraso^a^, Hills
Rational thinking style	Swami
Affect infusion model	Krekel
Mood maintenance model	Krekel
Dual processing model	Gould

### Psychological Associations with Behaviour

One hundred and twenty-nine psychological constructs were measured to determine if there was an association with TRBs/intentions. The TDF domains ‘social influences’ and ‘beliefs about consequences’ were the most investigated behavioural theoretical domains (listing 42 and 35 constructs and investigated by 17 and 15 studies, respectively) (Supplementary File 1 Table S5). No studies investigated the theoretical domains ‘reinforcement’ or ‘optimism’. Only the ‘intentions’ domain was consistently positively associated with adherence to TRBs with all five studies reporting an association with adherence to TRBs (Table 5). Intention was associated with physical distancing [18–20], hand hygiene [18–20], face covering [18, 20] and leaving the house (e.g. only leaving the house for permitted reasons such as essential shopping) [19–22]. There was no clear pattern of association between any of the other TDF theoretical domains and adherence to TRBs/intentions. One reason for a lack of a clear and consistent pattern is that studies investigated different constructs categorised under the same theoretical domain and found that some constructs were positively associated with TRBs/intentions and others were not. Another reason is that studies found that a construct was associated with some behaviours but not others. We illustrate the inconsistent pattern of results by reporting the results of those studies that were categorised under the TDF theoretical domain ‘social influences’, which was one of the most investigated domains. Further details about associations between theoretical constructs and TRBs are available in Supplementary File 1 Table S6.

**Table 5:** Number of studies reporting an association between a TDF theoretical domain and TRBs/intentions

	**Number of studies**		
**Theoretical domain**	**Investigating a construct within the theoretical domain**	**Reporting an association with intention/behaviour**	***Not* reporting an association with intention/behaviour**^**a**^
1. Knowledge	7	5	4
2. Skills	3	3	1
3. Social/professional role and identity	7	3	6
4. Beliefs about capabilities	10	10	7
5. Optimism	0	-	-
6. Beliefs about consequences	15	14	9
7. Reinforcement	0	-	-
8. Intentions	5	5	0
9. Goals	1	1	0
10. Memory, attention and decision processes	2	1	
11. Environmental context	6	3	5
12. Social influences	17	13	10
13. Emotion	4	4	0
14. Behavioural regulation	2	1	1
• Personality	2	2	0

• Personality is not one of the fourteen TDF domains, and so we have added it as a domain

^a^The number of studies reporting and not reporting an association with behaviour may not sum to the total number of studies investigating a construct in the domain because a study may report that a construct was associated for one behaviour but not for another behaviour or had measured several constructs that were categorised under the same domain and one construct was associated with behaviour/intentions and another was not

#### Illustration: Social Influences

Several studies examined the relationship between *descriptive/behavioural norms* (others engage in the behaviour) and adherence to TRBs but the evidence is inconsistent. One study found no association between behavioural norms and physical distancing, leaving the house and hand hygiene [36] while another found an association between descriptive norms and face covering but not for physical distancing and hand hygiene [18]. For *injunctive norms* (others approve of the behaviour), while two studies found no association with physical distancing, leaving the house and hand hygiene [36, 39], another found that beliefs about friends and family disapproval were associated with the behaviour leaving the house in people who had Covid-19 symptoms in the household but not if the household had no symptoms [23], and yet another study found associations with leaving the house to meet family and friends but not for other reasons [25]. A related study found injunctive norms were related to intention to leave the house but not to the actual behaviour [28].

Few studies investigated *social support*. Three studies from the same project found that people’s perceptions of support from a special person and family was not associated with self-isolating and leaving the house [21, 22, 24], but perceptions of support from friends [22] and community [21] were associated: people with lower perceived social support from friends and the community were more adherent [21]. People giving help to someone outside of their household were less adherent to TRBs than those not giving help [25], while people receiving help from someone outside their household [26] were less likely to leave the house. One study found that people with more *social contacts* and lower loneliness were more adherent to TRBs [25].

More studies than not found that *trust in public institutions* was associated with behaviour/intentions. People with higher trust in science [27], confidence in government [21, 25, 28] and confidence in the health system [25] were more adherent than people who had lower trust in the TRBs self-isolating, leaving the house, physical distancing and hand hygiene. However, in two studies from the same project [22, 24], trust in government was not associated with self-isolating and leaving the house behaviours. Trust in government was also associated with intentions to get Covid-19 testing but not with the behaviour leaving the house [29]. Another study found that people’s perceptions of being supported, well-informed and taken seriously by the government were not associated with adherence to government instructions [30]. Two studies investigated belief in the *credibility* of sources of information; it was associated with greater likelihood of wearing a face covering [31] but not with adherence to government instructions relating to Covid-19 protection behaviours [30]. One study found that people with more *social contacts* and lower loneliness were more adherent to TRBs [25].

In sum, some behaviours were associated with some indices of social influence but there was no consistent pattern over different constructs or different behaviours and patterns were different for people with different social contacts or experience of Covid-19.

## Discussion

### Key Findings

In total, 123 different TRBs were measured in 35 UK studies. TRBs most frequently investigated during the first wave of the pandemic were two avoidant behaviours: social gatherings and leaving the house, and two preventive behaviours: hand hygiene and face covering. Investigation of these behaviours is not surprising because these behaviours were either mandated (not leaving the house during lockdown except for essential reasons such as shopping) or recommended (hand hygiene) by UK governments, and it was not until 30 December 2020 that the Medicines and Healthcare products Regulatory Agency approved the Oxford AstraZeneca vaccine for use in the UK. The majority were UK-wide studies with seven studies explicitly referring to the inclusion of participants in all four nations. Given that health policy is a devolved issue then research conducted in all parts of the UK was necessary and recognised by UK behavioural scientists.

The two most commonly investigated sociodemographic variables were age and gender. Fifty-seven percent of studies included a measure of age and gender and 31% a measure of ethnicity, highlighting gaps in evidence of population-level adherence to TRBs. Half of the studies that included these variables found an association with behaviour/intentions. It is uncertain why findings are equivocal and why some studies investigating age and gender produced null findings, but it may be because the relationship varies by the type of behaviour or it may be due to sampling bias or how the behaviour is defined and measured. Studies conducted in other countries have found a positive association between female gender and older age and adherence to TRBs during the Covid-19 pandemic [32–39] and previous pandemics [40]. These sociodemographic differences in behaviour may be explained by gender- and age-related differences in the psychological predictors of behaviour during the Covid-19 pandemic. A study conducted in the USA, for instance, found that older people expressed higher trust in government Covid-19 information sources compared to young people [41]. Similarly, a study conducted in Pakistan found that risk perception was higher in females and that more females adhered to government rules than men [42].

The majority of studies used a cross-sectional study design, commissioned a marketing research company that applied the method of non-probability quota sampling and investigated self-reported behaviour. These characteristics of studies are likely a reflection of the need to set up and conduct studies as rapidly as possible in order to produce evidence that could inform policy. A limitation of the cross-sectional study design is that because TRBs and predictor sociodemographic and psychological variables are simultaneously assessed, it is not possible to establish a true cause and effect relationship. Self-reported behaviour also has limitations; a recent systematic review found self-report over-estimating observed adherence of TRBs by up to a factor of five in some settings [43].

The two most common behavioural theories used were Protection Motivation Theory and the Theory of Planned Behaviour/Reasoned Action Approach. In total, 129 different psychological constructs were measured. The two most common domains investigated from the TDF were ‘social influences’ and ‘beliefs about consequences’. A scoping review of 84 international studies about predictors of ‘social distancing’ behaviour also applied the TDF and found that ‘environmental context and resources’ was the most coded theoretical domain and that the construct most frequently coded under this domain was ‘personality trait’ [44]. The scoping review categorised sociodemographic constructs under the environment theoretical domain. While sociodemographic constructs highlight *who* adheres, only psychological constructs can explain *why* people adhere, which is why we chose not to categorise sociodemographic variables as a behavioural theoretical domain. However, both reviews suggest that there are important gaps in the theoretical domains measured, including’optimism’, ‘goals’, ‘reinforcement’ and ‘behavioural regulation’. Lack of measurement of constructs within these theoretical domains means our understanding of the role of these constructs in adherence to TRBs during the pandemic within the UK context is limited.

Our review suggests that there is consistent evidence (from five studies) that people with higher intentions to perform a behaviour reported performing a behaviour compared to people with lower intentions. Therefore, this review and studies conducted elsewhere [45] found evidence of a positive association between intentions and behaviour. This confirms decades of previous research that has demonstrated a relationship between intentions and behaviour; a meta-analysis of 10 previous meta-analysis (422 studies in total) for example, found a large sample-weighted average correlation between intentions measured at one time-point and measures of behaviour taken at a subsequent time-point (r_+_ 0.53) [46].

This current review of UK data together with the broader international literature have found that people’s beliefs about the Covid-19 disease and about the TRBs themselves predict behaviour [32, 36, 47–49]. Studies reported in this review show that the UK general public were motivated to change their behaviour because they wished to avoid the negative consequences of the Covid-19 disease on themselves, their family and friends and wider society and believed that the consequences of actually performing preventive and avoidant behaviours would be beneficial. Hence, beliefs about disease and about behaviour were important motivational factors during this, as well as previous pandemics [40].

The review found that there is inconsistent evidence that ‘social influences’ are associated with behaviour but there is no clear explanation for these inconsistencies; it may be due to sampling bias and how different constructs were defined and measured. However, more studies than not found a positive association between people’s trust in public authorities and behaviour adherence, and more studies than not found that people’s beliefs about the consequences of the Covid-19 disease and their beliefs about the consequences of the behaviours for self, family and friends, and wider society were positively associated with behaviour/intentions. The results of studies about the relationship between control beliefs and behaviours / intentions were inconsistent.

Beliefs about the Covid-19 disease (e.g. Covid-19 is very contagious) and beliefs about the effectiveness of behaviours to prevent disease transmission and severity are, however, not sufficient to change behaviour. Beliefs alone do not inevitably translate into actual behaviour change; that is, people intend to act, but fail to realise their intentions (this is typically referred to as the ‘intention-behaviour gap’ [50]). The Health Action Process Approach (HAPA) conceptualises beliefs as pre-intentional motivational processes that contribute to behavioural intention and represent proximal and distal determinants for developing an intention to change behaviour [51]. The HAPA proposes a volitional phase, when a person’s intention is translated into actual behaviour via self-regulatory strategies (i.e. behavioural regulation) such as action planning and goal-setting. This review however, found a gap in evidence; while UK behavioural scientists have advanced understanding about what beliefs predict behaviour during the first two waves of the pandemic in the UK, there is a notable lack of evidence regarding the TDF theoretical domain ‘behavioural regulation’. Studies conducted elsewhere have investigated this theoretical domain; a study conducted in Belgium for instance found a positive association between action planning and behaviour adherence during the current pandemic [45]. This review also found lack of evidence about the TDF theoretical domain ‘reinforcement’, which includes incentives and punishments and sanctions. Yet, in the UK, a legal duty to self-isolate came into force during the second wave of the pandemic with fines for those breaking the rules starting at £1000 and increasing up to £10,000 for repeat offenders (the punishment incentive) in contrast to the incentive of £500 for people on lower incomes who have lost income as a result of having to self-isolate [52]. Studies conducted elsewhere suggest that intention to self-isolate was associated with beliefs about financial compensation [53]. Clearly, reinforcement processes played a role in TRBs such as self-isolation, but this was not examined within the academic literature.

## Limitations of the Review

This review was conducted in line with an a priori protocol, and we provide sufficient detail in this manuscript for the purposes of transparency and replicability. However, there are a number of limitations which constrain our ability to draw firm conclusions about determinants of behaviour. We relied on electronic databases to identify studies which means that there is a risk of publication bias although we did include non-peer-reviewed articles published in PsyArXiv. We appraised the quality of evidence, highlighting risk of sampling bias and treated all evidence as valid for the purposes of the narrative synthesis. The heterogeneity in the predictors and TRBs measured and the measures used to assess predictors and TRBs precluded meta-analyses that could have confirmed or refuted the conclusions drawn from our narrative synthesis. Internationally agreed definitions and methods for categorising pandemic behaviours, sociodemographic and psychological constructs are required so that the findings of individual studies and evidence synthesises can be directly compared, thereby making behavioural research easier to translate into public health policy and practice. We used the TDF to classify psychological constructs, but research teams vary in how these constructs are interpreted, investigated and classified. Similarly, we encountered several challenges when comparing the findings of individual studies because of the application of different definitions and measures for both sociodemographic and psychological variables for example, the use of different age bands and measures of risk perception. This review includes UK studies primarily conducted in the first wave of the pandemic and therefore may not be relevant to current and future waves or other countries. Nonetheless, some findings corroborate behavioural theory as well as pre-pandemic research conducted about other behaviours.

## Conclusions: Implications for Future UK Public Health Interventions

There is a need for rapid interventions to support people to maintain their preventive and avoidant behaviour during pandemics. These interventions are best informed by theory-based empirical studies. Despite some of the limitations of the 35 included studies, this review suggests that in the UK some people may increase adherence from interventions designed to shift beliefs about the Covid-19 disease and about the effect of behaviours on self, family and society. Research teams need to address important gaps in evidence such as lack of understanding of behavioural regulation, the effectiveness of reinforcement strategies on behaviour and study designs better able to support causal inferences. Behavioural scientists may have more direct impact on public health policy and practice if there was consensus on definitions, methods and a core set of outcome measures so that a meta-analysis of psychological determinants of behaviour during a pandemic that is actionable can be presented to public health agencies. There is a greater need for academic behavioural scientists and public health agencies to formally collaborate. Building relationships now may help us be better prepared for future pandemics.

### Supplementary Information

Below is the link to the electronic supplementary material.

Supplementary file1 (DOCX 153 KB)

## Declarations

### Welfare of Animals

NA.
