# Supplementary material for: Behavioural Sciences Contribution to Suppressing Transmission of Covid-19 in the UK: A Systematic Literature Review
Source: Int J Behav Med. 2023 Apr 14;31(1):1–18. doi: 10.1007/s12529-023-10171-4 (PMC10104693; doi:10.1007/s12529-023-10171-4)
Supplement: Supplementary file 1 — Supplementary file1 (DOCX 153 KB) [file 12529_2023_10171_MOESM1_ESM.docx]

# Table S1: Eligibility criteria

|  | **Inclusion** | **Exclusion** |
| --- | --- | --- |
| 1 | UK general population or sub-groups of the general population e.g. older people | Patients with a medical condition e.g. patients with heart disease, healthcare workers, university students |
| 2 | General population settings | Specific settings e.g. care homes, hospitals, schools |
| 3 | (1) Levels of adherence to Covid-19 preventive behaviours performed by individuals e.g. physical distancing, hand hygiene, wearing a face covering, testing, self-isolation/quarantine, and/or  (2) Associations between psychosocial and/or sociodemographic variables and behaviour / intention | Other behaviours e.g. help-seeking  Shielding  Vaccination |
| 4 | Any study design e.g. cross-sectional, prospective, controlled trials / experiments but only if analyses of levels of adherence and /or associations between psychosocial and sociodemographic variables and behaviour / intention is reported | Epidemiological modelling studies  Qualitative studies  Opinion pieces and commentary  Literature reviews  Protocols  Service evaluation |
| 5 | United Kingdom (Wales, Scotland, Northern Ireland, and/or England) study or part of an international study | If UK data were not reported separately |

# Table S2: Assessment of sampling bias

| **Author** | **Sample Size** | **Q1: Was the sample size justified?** | **Q2: Was the target/reference population clearly defined? (is it clear who the research was about?)** | **Q3: Was the sample frame taken from an appropriate population base so that it closely represented the target/reference population under investigation?** | **Q4: Was the selection process likely to select subjects/participants that were representative of the target population under investigation?** | **Q5: Were measures taken to address and categorise non-responders?** | **Q6: Does the response rate raise concerns about non-response bias?** | **Q7: If appropriate, was information about non-responders described?** | **Q8: Was the sample achieved reported as broadly representative of the UK population?** | **Q9: Was it reported that data were weighted to ensure analyses properly reflected the UK population?** | **Q10: Were characteristics of responders included and not included in the analysis compared?** |
| --- | --- | --- | --- | --- | --- | --- | --- | --- | --- | --- | --- |
| **Armitage**  [1] | 2252 | No | Yes - UK adults ≥18 yrs | YouGov, a market research company, recruited a sample from their existing database | A sample of adults designed to be representative of the UK population was invited to take part. | No | Yes - response rate not reported | No | The sample achieved was broadly representative of the UK population. | Yes | No |
| **Atchison** [2] | 2108 | No | Yes - UK adults ≥18 yrs | YouGov | A sample of 2108 adults was achieved through non probabilistic quota sampling. Emails were sent to 2500 panellists from the base sample, randomly selecting panelist with particular age, sex, ethnicity and UK geographical region of residence characteristics to achieve quotas  that matched the proportions of people with those characteristics in the UK 2011 census data | No | No – 84.3% response rate | No | There was lower response among people from minority ethnic groups and older age groups compared with the UK population  profile | Yes | No |
| **Bacon** [3] | 202 | No | No | Prolific – an online research participants’ platform, representative of the general population | Not described | No | Yes – not reported | No | not reported | No | No |
| **Bowman** [4] | 2768 | No | Yes - UK adults ≥18 yrs | YouGov | UK sample was obtained through a nonprobabilistic active sampling method, and emails were sent to randomly selected individuals with particular characteristics to match the proportions of people with those characteristics in the 2011 UK | No | No – 84.3% response rate | No | not reported (but it’s the same sample as Atchison) | Yes | No |
| **Dixon** [5] | 2969 | No | Yes - Adult men and women aged 16 or older, able to speak English, and currently living in Scotland were eligible to participate | Ipsos MORI Scotland). MORI conducted telephone interviews using computer-aided telephone interviewing (CATI), which  involves random digit dialling to both landlines and targeted mobiles. | Quotas were applied to ensure a representative sample of Scotland adults was achieved. Quotas were based on gender, age, working status, and geographical locations (distribution over the Scottish Parliament regions). | No | Yes – response rate not reported | No | Representativeness was assessed against four criteria - gender, age, working status and Scottish Parliament regions – and was achieved | No | No |
| **Dowthwaite** [6] | 1001 | No | Yes – 16 – 75 year olds | Ipsos MORI | Ipsos MORI via email to a nationally representative sample, based on age, gender, and region, drawn from a randomly selected pool of participants who met the relevant criteria. There was also a 10% to 15% quota for BAME respondents, with the same process applied to ensure hitting the minimum required quota. As fieldwork progressed, they specifically targeted any quota groups that were still required to meet the final profile that was needed, again randomly selecting within those groups. A total of 2575 invitations to take part in the study were sent out. | No | 39% response rate (1001 respondents) |  | not reported | Yes | No |
| **Eraso (b)**^1^ [7] | 681 | Yes - In order to achieve a 99% confidence level and 5% margin of error in relation to the 1,777,666 population of the qualifying boroughs a minimum sample size of 663 was required | Yes -   18 years and a resident in the London boroughs | a link to  the questionnaire was disseminated via the authors’ university website and social media accounts, local newspapers, and North London Facebook community groups. | There was no selection process | No | Yes | No | Of note is that the restricted sample was highly skewed to females, with 82.7% of respondents being female (211 vs. 42 males). Additionally, of note is that a minority of 13.7% of the restricted sample  came from Black, Asian and Minority Ethnic (BAME) populations (35 vs. 220 White), which is disproportionate to the 40.2% of the broader London population who come from BAME groups | No | No |
| **Eraso (a)**^1^ [8] | 681 | Yes - See Eraso (b) | See Eraso (b) | See Eraso (b) | See Eraso (b) | See Eraso (b) | See Eraso (b) | See Eraso (b) | See Eraso (b) | See Eraso (b) | No |
| **Fujii** [9] | 994 | No | UK adults ≥18 yrs | Market  research company Lucid. | Individuals  were initially contacted via email to participate in the online survey. New invitations were sent up to the point where representativeness was achieved on age, gender and household income | No | No | No | The participants  are confirmed to be nationally representative in  terms of age structure, sex, and household income in  each country. | No | No |
| **Galasso** [10] | 2012 | No | No | No | No | No | No | No | Not reported | No | No |
| **Hills**^1^ [11] | 681 | Yes - See Eraso (b) | See Eraso (b) | See Eraso (b) | See Eraso (b) | See Eraso (b) | See Eraso (b) | See Eraso (b) | See Eraso (b) | See Eraso (b) | No |
| **Jain** [12] | 130 | NA | Yes - Existing London coronavirus Response Centre records | All COVID-19 cases attending workplaces in the 2 days prior to symptom onset (i.e. during their infectious period) were initially identified through tier two of NHS Test and Trace as part of the contact tracing process, and escalated to the LCRC for further management | All Covid-19 cases reported to LCRC | NA | NA | NA | NA | NA | NA |
| **Jorgensen** [13] | 904 | No | No | Epinion sampled adult respondents using online panels. | To  increase representativity survey respondents were quota-sampled to match the population  margins on age, gender, and geographic location | No | Response rate not reported | No | our study are skewed towards more educated and younger eligible voters compared  to the overall population of eligible voters. In contrast, the samples are overall well balanced on  sex. | No – instead we address these imbalances by controlling for a battery of  covariates that match these imbalances | No |
| **Keyworth** [14] | 2252 | No | No | A representative  sample of the UK population were invited to take part in an online  questionnaire, administered as part of a daily “omnibus” survey | Don’t know | No | Don’t know | No | Don’t know | Yes | No |
| **Lawson** [15] | 498 | No | Yes | NA | Yes | NA | NA | NA | NA | NA | NA |
| **MacIntyre** [16] | 300 | Yes - The study was powered a priori to identify a 20% difference in the rate of mask use between cities with and without mask mandates with 95% confidence and 80% power. A mask use prevalence of 60% was assumed among cities without mask mandate and 80% among cities with mask mandate (Babalola et al., 2020; Jones, 2020) and a sampling ratio of 0.3, yielding a minimum required sample size of 194. Hence, this study aimed to recruit a total of 2150 participants, from which the samples were selected proportionate to population size, age and gender distribution f the sampled cities(i.e. 300 from London). Post-hoc power analysis was conducted via large sample approximation using G*Power 3.1.9.7 (Faul et al., 2009). In a two-sided test with α _= 0.05, the recruited sample size (N = 2343) enabled detection of at least 20% difference in mask use (i.e. odds ratio of 1.2), yielding a power of 94.1%. | Yes | A market research company Dynata (Dynata, 2020), randomly distributed the survey link by email to a representative sample of their panel members | Don’t know | No | No | No | Don’t know | No | No |
| **Maher** [17] | 272 | Yes - we estimated that at least 200 participants would be  required to visualize opinion-based groups. To accommodate longitudinal attrition, we  aimed for 300 participants at Time 1 (T1). | No | Prolific | Don’t know | No | No | No | Don’t know | NA | No |
| **Margraf** [18] | 1105 | No | Yes | YouGov | Yes | No | Don’t know | No | Don’t know | No | No |
| **Norman** [19] | 477 | No | Yes | Prolific | Prolific uses quota sampling  to recruit samples from their pool of research participants that are broadly representative  of the UK adult population in terms of age, sex, and ethnicity | No | No | No | Don’t know | No | Attrition analyses revealed that those who did not complete all of the  measures (N = 23) were younger (M = 34.52, SD = 15.54 vs. M = 24.22, SD = 15.20, t  (498) = 3.60, p < .001) and had lower capacity scores for not visiting family and friends  (M = 6.13, SD = 1.55 vs M = 6.56, SD = 0.97, t(498) = 2.01, p = .045) than those who  completed all of the measures (N = 477). |
| **Perrotta** [20] | 8753 | No | Yes | Don’t know | Don’t know | No | Don’t know | No | The sex ratio is somewhat skewed towards women compared to the overall population,  Furthermore, older  adults tend to be over-represented | Yes | No |
| **Schneider** [21] | 6281 | No | Yes | Prolific | provided national quota samples stratified by age,  gender, and ethnicity | No | Don’t know | No | Don’t know | No | No |
| **Schuz** [22] | 477 | No | Yes | Prolific | Quota sampling was used to recruit participants  from the pool of individuals signed up to Prolific who were roughly  representative of the UK adult population in terms of age (18–24: 12.0 %  (UK)/12.4 % (study sample), 25–34: 17.0 % (UK)/17.0 % (study sample),  35–44: 17.7 % (UK)/17.4 % (study sample), 45–54: 17.6 % (UK)/  18.0 % (study sample), 55+: 35.7 % (UK)/35.2 % (study sample); Office  for National Statistics, 2020b), sex (females: 50.6 % (UK)/51 % (study  sample); Office for National Statistics, 2020b) and ethnicity  (non-white:15 % (UK)/18 % (study sample); gov.uk, 2020). | No | No | No | Quota sampling was used to recruit participants  from the pool of individuals signed up to Prolific who were roughly  representative of the UK adult population in terms of age (18–24: 12.0 %  (UK)/12.4 % (study sample), 25–34: 17.0 % (UK)/17.0 % (study sample),  35–44: 17.7 % (UK)/17.4 % (study sample), 45–54: 17.6 % (UK)/  18.0 % (study sample), 55+: 35.7 % (UK)/35.2 % (study sample); Office  for National Statistics, 2020b), sex (females: 50.6 % (UK)/51 % (study  sample); Office for National Statistics, 2020b) and ethnicity  (non-white:15 % (UK)/18 % (study sample); gov.uk, 2020). | No | The retained sample were older than those omitted (M = 34.52, SD =  15.54 vs. M = 24.22, SD = 15.20, t(498) = 3.60, p < .001) but otherwise  similar on measured variables. |
| **Shiina** [23] | 2000 | No - As this was an exploratory study, no specific hypothesis  was tested. Therefore, we could not calculate the  minimum required sample size. However, an estimate of  500 samples from each region is considered adequate for  interregional comparison studies, and multivariate analysis  requires generally ten-fold of the number of variables  After consideration of the size of the research budget,  we chose to obtain 2000 samples from the UK | Yes | Cross Marketing Group Inc. | Don‘t know | No | Don ‘t know | No | Don’t know | No | No |
| **Smith (a)** [24] | 2240 | No - We calculated achieved power for the analyses (in households  with and without symptoms) using post-hoc power calculations | Yes - aged 18 years or older and living in the UK. | YouGov | Quota sampling was  used, based on age, gender, social grade, level of education and  Government Office Region, to ensure that the sample was broadly  representative of the UK general population. | In total, 74 participants  were excluded because of a lack of data for sociodemographic  variables, suspiciously fast completion of the survey or providing  identical answers to multiple consecutive questions. | No | In total, 74 participants  were excluded because of a lack of data for sociodemographic  variables, suspiciously fast completion of the survey or providing  identical answers to multiple consecutive questions. | Don’t know | No -Weighting data by age, gender, social grade, highest level of  education and region altered prevalence of outcome behaviours  only slightly. We therefore used unweighted data in our analyses. | No |
| **Smith (b)** [25] | 6149 | Yes - A sample size of 6,150 allows a 95% confidence interval of plus or minus 1% for the prevalence  estimate for each survey item. | Yes | Predictiv’s research panel | Quota sampling, fulfilling pre-specified targets based on age, gender, income and region, was  used to ensure the sample that was broadly representative of the general UK population.  This method of sampling allows proportionate representation of target groups in the sample  that may otherwise be under-represented (e.g. older adults). | No | 89% of people who clicked on the  link subsequently completed the study materials. | No | Don’t know | No - Our analyses report unweighted statistics. | No |
| **Smith (c)** [26] | 53880 | Yes - We determined that a sample size of 2000 in each  survey would allow a 95% confidence interval of plus  or minus 2% for the prevalence estimate for a survey  item with a prevalence of around 50%. In practice,  power was considerably better as we pooled data from  survey waves. | Yes | BMG Research, a Market Research Society company  partner, | As prompt turnaround of data  collection is essential during a rapidly evolving crisis,  the surveys used standard opinion polling methods  using non-probability sampling, an approach common  within market research, political polling, and social  science. Quota samples aim to minimise response  bias by filling predetermined targets so that the  social and personal characteristics of the participants  match those of the national population. As such,  participants who belong to a quota that has already  been met are prevented from completing the survey.  Therefore, response rates are not useful indicators of  response bias in quota samples and are not usually  reported. | No | No | No | Yes - Quotas  were applied based on age and sex (combined) and  government office region and reflected targets based  on data from the Office for National Statistics.  Therefore, the sociodemographic characteristics of  participants in each survey wave were broadly similar  to those in the UK general population. | No | No |
| **Swami** [27] | 520 | No | Yes | Prolific website, a crowdsourcing internet  marketplace that allows individuals to complete academic  surveys for monetary compensation, on April | Cross-stratified quota sampling was used to obtain a  nationally representative sample of the adult (>18  years) UK population based on gender identity, age,  and ethnicity profiles at the last census. | No | Don’t know | No | Don’t know | No | No |
| **Woelfert** [28] | 301 | Yes - Based on an a priori power simulation using the *SIMR* package 300 participants would provide 90% to observe a small to medium effect at a 0.05 significance level. | No | Prolific. | Don’t know | No | No | No | Don’t know | No | No |
| **Wright (a)**  [29] | 22625 | No | Yes | The sample was recruited using three primary approaches. First,  snowballing was used, including promoting the study through existing  networks and mailing lists (including large databases of adults who had  previously consented to be involved in health research across the UK),  print and digital media coverage, and social media. Second, more targeted  recruitment was undertaken focusing on (i) individuals from a  low-income background, (ii) individuals with no or few educational  qualifications, and (iii) individuals who were unemployed. Third, the  study was promoted via partnerships with third sector organisations to  vulnerable groups, including adults with pre-existing mental health  conditions, older adults, carers, and people experiencing domestic  violence or abuse. | The sampling is not random and therefore is not  representative of the UK population, but it does contain a heterogeneous  sample. | No | No | No | Don’t know | Yes | describe who included in analysis - When comparing descriptive statistics and  compliance levels according to last month of data collection in the study,  participants who were included in the main analysis (i.e. data collection  up to August 2020) had higher and less steep drops in compliance, were  older, less likely to be employed, and differed on several personality  traits, including higher optimism and resilience and lower neuroticism  and openness to experience |
| **Wright (b)** [30] | 51600 | No | Yes | As above | As above | No | No | No | Don’t know | The weighted data were matched to population  statistics across the following characteristics: age, gender, ethnicity,  education, and country of living. Population statistics were taken  from the ONS’s Annual Population Survey | No |
| **Juanchich** [31] | 1105 | No | No | Prolific Academic platform | Don’t know | No | Don‘t know | No | Don’t know | No | No |
| **Krekal**  [32] | 30000 | No | No | University College London Covid-19 Social Survey | Don’t know | No | Don‘t know | No | Don’t know | No | No |
| **Raihani** [33] | 739 | No | No | Prolific Academic | Don’t know | No but - We used the website www.getipintel.net to identify bot, proxy and VPN submissions to our 97 survey. As recommended, we excluded any submission from a participant where the probability of the IP Address being fraudulent was 0.99 or more. This resulted in 11 exclusions from the UK data. We subsequently removed data points from any participant who completed the entire survey in less than 200 seconds to exclude participants who were not paying sufficient attention to the questions (13 subjects excluded from UK wave 1 sample). Our key results are robust to these exclusions. We finally reduced the dataset such that we had complete responses for all participants for all categories included in our analyses. For wave 1, we therefore had complete responses from 698 UK participants | No | No | Don’t know | No | No |
| **Lewis** [34] | 1011 | No | Yes | Participants were sampled from the NHS Wales Test, Trace, Protect (TTP) service using quota sampling based on age and gender (interlocked) and Welsh Index of Multiple Deprivation quintile to ensure the sample was representative of contacts informed to self-isolate during this period. | Participants were sampled from the NHS Wales Test, Trace, Protect (TTP) service using quota sampling based on age and gender (interlocked) and Welsh Index of Multiple Deprivation quintile to ensure the sample was representative of contacts informed to self-isolate during this period. | A response rate of 19.9% achieved 1,011 completed telephone interviews. Reasons for non-participation included no answer to the telephone (N=3,020, 59.3%), refusal to participate (N=659, 12.9%), an inconvenient time (N=271, 5.3%), an invalid phone number (N=69, 1.4%), contact currently self-isolating (N=46, 0.9%), not notified as a contact (N=9, 0.2%), and duplicate (N=7, 0.1%). | A response rate of 19.9% achieved 1,011 completed telephone interviews. Reasons for non-participation included no answer to the telephone (N=3,020, 59.3%), refusal to participate (N=659, 12.9%), an inconvenient time (N=271, 5.3%), an invalid phone number (N=69, 1.4%), contact currently self-isolating (N=46, 0.9%), not notified as a contact (N=9, 0.2%), and duplicate (N=7, 0.1%). | No | Don‘t know | Yes - Weights were applied to all analyses to ensure that the final sample was representative of all eligible contacts of COVID-19 in Wales drawn from the TTP database. | No |
| **Gould** [35] | 580 | No | Yes | NA | Yes | NA | NA | NA | NA | NA | NA |

# Table S3: Studies reporting behaviour and / or intention and associations between sociodemographic and psychological factors

| **Author** | **Behaviour / intentions** | | **Associations with behaviour / intentions** | |
| --- | --- | --- | --- | --- |
|  | **Behaviour** | **Intention^1^** | **Sociodemographic** | **Psychological** |
| Armitage | x |  | x | x |
| Atchison | x | x | x |  |
| Bacon |  | x | x | x |
| Bowman | x^2^ |  | *UK data not reported separately* | x^2^ |
| Dixon | x |  | x | x |
| Dowthwaite | x | x | x |  |
| Eraso (b) |  | x | x | x |
| Eraso (a) | x |  | x | x |
| Fujii | x |  | x | x |
| Galasso | x^2^ |  | x^2^ | *UK data not reported separately* |
| Hills | x |  | x | x |
| Jain | x |  | x |  |
| Jorgensen | x |  |  | x |
| Keyworth | x |  |  |  |
| Lawson | x |  |  |  |
| MacIntyre | X^2^ |  | *UK data not reported separately* | *UK data not reported separately* |
| Maher | x |  | x | x |
| Margraf | x |  | x | x |
| Norman | x |  | x | x |
| Perrotta | x |  |  |  |
| Schneider | x |  |  | x |
| Schuz | x | x | x | x |
| Shiina | x |  |  | x |
| Smith (a) | x |  | x | x |
| Smith (b) | x |  | x |  |
| Smith (c) | x | x | x |  |
| Swami | x |  |  | x |
| Woelfert | x |  | x | x |
| Wright (a) | x |  | x | x |
| Wright (b) | x |  |  | x |
| Juanchich | x | x | x | x |
| Krekel | x | x |  | x |
| Raihani | x |  | x | x |
| Lewis |  | x |  | x |
| Gould | x |  |  |  |

^1^ Measured as a dependent variable ^2^ UK data reported separately and therefore could be extracted

# Table S4: Results of all studies that investigated associations between gender and/or age and behaviour/intention

| **Study** | **Behaviour** | **Which gender was more adherent?** | **Age groups^1^** | **Which age was more adherent?** | **Which ethnicity group was more adherent?** | **Which education group was more adherent?** | **Which employment group was more adherent?** |
| --- | --- | --- | --- | --- | --- | --- | --- |
|  | **Adherence to rules and regulations** |  |  |  |  |  |  |
| Armitage | How closely following the UK government’s COVID-19-related instructions | Females |  | Older people | White ethnic background | - | No differences |
| Margraf | How much adhere to rules | Females | Five groups:  18-24  25-34  35-44  45-54  55 years and older | Older people |  |  |  |
| Wright (a) | If following the recommendations from authorities to prevent spread of Covid-19 | Females | Four groups:  18-29  30-45  46-59  60+ | All ages groups over 29 | No difference | Poorer education | Retired |
|  | **Hand hygiene** |  |  |  |  |  |  |
| Dixon | How often practiced hand hygiene (aggregate of 3 items: washing hands as soon as you get home; washing hands using soap and water; washing hands for at least 20 s and washing hands before eating and drinking) | Females | Five groups:  1-24  25-34  35-44  45-54  55-64 | More 45-54 and 55-64 compared to other age groups | No difference |  | No difference |
| Fujii | How often engaged in handwashing with water and soap or using hand sanitizer | No difference | Two groups:  Under 65  65 and over | No difference |  |  |  |
| Lawson | - Adequate hand hygiene: this involved washing hands with water, soap and then lathering for twenty seconds or more and scrubbing in various rotations and interlocking of fingers, after which hands are rinsed with water to remove soap excess and then dried properly using an appropriate drying method for fifteen seconds or more - Basic hand hygiene: this involved washing hands with water, soap and scrubbing hands in various rotations and interlocking of fingers after which hands are rinsed with water to remove soap excess, and then dried afterward using an appropriate drying method but not for the recommended minimum length of time - Poor hand hygiene: this involved any other combination of steps not fitting the two previous categories - Non-hand hygiene: this involved not washing or drying hands at all. | No difference  More females  No difference  Less females | Two groups:  Children  Adults | More children  More adults  Less children |  |  |  |
| Norman | How often not washed hands as soon as got home | Females |  | Older people | No difference |  |  |
|  | **Physical distancing** |  |  |  |  |  |  |
| Dixon | Physical distancing (1 item: staying 2 m (6 feet) away from other people.) | No difference | See above | More 45-54 and 55-64 compared to other age groups | No difference |  | Not working full-time |
| Norman | Keep > 2 metres outside  Keep > 2 metres inside | No difference  No difference |  | Older people  No difference | No difference |  |  |
|  | **Face covering** |  |  |  |  |  |  |
| Dixon | Face covering (2 items when in a shop and when travelling on public transport) | No difference | See above | More 45-54 and 55-64 compared to other age groups | No difference |  | Not working full-time |
| Fujii | Wearing a mask | No difference | See above | More over 65 years olds |  |  |  |
|  | **Self-isolation** |  |  |  |  |  |  |
| Atchison | *Willingness to self-isolate* | Females |  | No difference | No difference | educated to degree level | No difference |
| Bacon | *Intend to self-isolate* | No difference |  | Younger people |  |  |  |
| Eraso (b) | Sub-group analysis of those who had symptoms or lived with someone who had symptoms - were asked how many times they left their house and how many times someone visited as a measure of self-isolating | No difference |  | No difference | No difference | No difference | No difference |
| Smith (c) | Sub-group analysis of those who had experienced symptoms were asked how many times they left their house | Females |  | Older people | No difference | Poorer education | Not working in a key sector |
| Jain | Not attending workplace after symptom onset | Males | Three age groups:  18-29  30–49  ≥ 50 | No difference |  |  | No difference |
| Dowthwaite | *Intend to self-isolate* | - | Two groups:  Under 65  65 and over | Of those who did not have the app no difference. Of those with the app who had not been notified to self-isolate also no difference but of those who had not been notified, people 65 and over had higher intention to comply | Those with the contact tracing app, white ethnic groups greater intention to self-isolate  Those without the app no difference |  |  |
|  | **Social gatherings** |  |  |  |  |  |  |
| Fujii | Avoid social gatherings | No difference | See above | No difference |  |  |  |
|  | **Leaving home** |  |  |  |  |  |  |
| Norman | Limit leaving home  Not visit friends/family | No difference  No difference |  |  | No difference |  |  |
|  | **Testing** |  |  |  |  |  |  |
| Juanchich | *Intention to get tested if the government was recommending them to do so* | No difference | Four groups:  18-25  26-40  41-55  55+ | No difference |  |  | No difference |
| Smith (c) | Requesting a test if had symptoms  *Intention to share details of close contacts if tested positive* | Females  Females |  | No difference  Older people | No difference  No difference | No difference  Higher education | No difference  Working |
|  | **Aggregated scores for behaviour** |  |  |  |  |  |  |
| Atchison | (1) Aggregate for social distancing:  avoiding public transport  avoiding social events  avoiding going out in general  avoiding going to hospital or other healthcare settings  avoiding crowded places  avoiding contact with people who have a fever or respiratory symptoms | No difference | Six groups:  18-24  25-34  35-44  45-54  55-69  70 above | 70 years or older than younger adults aged 18-34 years | No difference | No difference | No difference |
| Eraso (a) | (1) Aggregate of:  Went out to meet up with family, went out to meet up with friends, went out for unpermitted reasons, went out for permitted reasons but unable to keep 2 metre physical distance (parks and public spaces for exercise, went out for groceries, went out for medication)  (2) Intentional infringement of social distancing rules to go out to meet up with family, go out to meet up with friends and go out for unpermitted reasons | No difference  No difference |  | No difference  Additional year of age increases intentional SD infringements by 1.7%. | No difference  No difference | No difference  No difference | No difference  No difference |
| Hills | As 818 Eraso because it is the same study with each article reporting some different results | No difference |  | No difference | No difference | No difference | No difference |
| Woelfert | Aggregate of:  avoid in-person contact with others  avoid social gatherings in person  try to keep a safe distance to others | No difference |  | No difference |  | No difference |  |
| Smith (a) | Aggregate of:  Left home to go to the shops for groceries, toiletries or medicine  Left home to go to the shops for other items  Left home for exercise  Left home for a medical purpose excluding going to the shops/pharmacy for medicine  Left home to go to work  Left home to help someone else  Left home to meet friends or family who they did not live with  (1) If have symptoms in household  (2) If don’t have symptoms in household | Females  Females | Five groups:  18-24  25-34  35-44  45-54  55 and older | No difference  55 and older |  | No difference  No difference | No difference  Not working |
| Galasso | Aggregate of:  keeping six feet distance from people outside home  not seeing friends  avoiding busy places  leaving home less than once a day  staying at home  shaking hands or hugging  face covering,  washing hands more often  coughing in elbow  wearing gloves | Females | - | - |  |  |  |
| Schuz | Aggregate of:  Only leave home for food shopping, exercise, medical needs or travelling to work (if you cannot work from home)  Keep at least 2 m (6 feet) away from other people when outside away from home  Keep at least 2 m (6 feet) away from other people when inside shops  Not visit or meet friends or other family members that you don’t live with  Limit yourself to one session of exercise (e.g. walk, run, cycle) close to home each day  Limit the number of times you leave home each week to shop for food  Wash your hands as soon as you return home  Wear a mask when away from home  *Intention to adhere* | Females  Females |  | Older people  No difference | No difference  No difference |  |  |
| Raihani | Aggregate of:  self-isolating,  working from home  staying at home whenever possible  avoiding social events  avoiding work events  reducing to stopping travel within the UK  reducing to stopping international travel  avoiding contact with vulnerable people  washing hands more frequently  trying to avoid touching face | No difference | Three groups:  18-34  35-54  55+ | No difference |  |  |  |
| Maher | Aggregate of: physical distancing and handwashing |  |  |  |  |  | No difference |

^1^ Unless age groups are specified, we assume that ag is analysed as a continuous variable

# Table S5: Psychosocial constructs included in analyses to determine associations with behaviour / intention categorised using the theoretical domains framework

| **Theoretical Domain** | **Constructs** | **Study** |
| --- | --- | --- |
| 1. Knowledge  (n=7 constructs; n= 7 studies) | Knowledge of the Covid-19 disease   1. Beliefs about causes of Covid-19 (causes) 2. Symptoms of Covid-19 are easy to recognise (identity) 3. Covid-19 a recurrent disease (timeline) 4. Knowledge about Covid-19   Knowledge of the behaviours   1. Knowledge about rules and guidelines 2. Communication by national govt and authorities clear and understandable 3. Understanding of government rules | Dixon  Dixon  Dixon  Wright (b)  Eraso (a), Eraso (b), Hills  Margraf  Smith (a) |
| 2. Skills (n=3 constructs; n=3 studies) | 1. COM-B physical capability (physical skills, strength to engage in behaviour) 2. COM-B psychological capability (knowledge and psychological skills to engage in behaviour) 3. Own past experience of the behaviour | Armitage  Armitage, Lewis  Schuz, Lewis |
| 3. Social / professional role and identity  (n=6 constructs; n=7 studies) | 1. Social responsibility values (e.g. consider needs of others) 2. Self-interest values (e.g. put own needs first) 3. Political party voted for 4. Political views / ideology 5. Beliefs about Brexit 6. Conspiracy beliefs 7. Covid-19 conspiracy theories | Eraso (a), Eraso (b), Hills  Eraso (a), Eraso (b), Hills  Eraso (a), Eraso (b), Hills  Maher, Juanchich, Raihani  Maher  Juanchich,  Juanchich, Swami |
| 4. Beliefs about capabilities  (n=9 constructs; n=10 studies) | Confidence   1. Self-efficacy / confidence to perform the behaviour / capacity 2. Response self-efficacy (confident can avoid disease by performing behaviour)   Control   1. Perceived personal control over the behaviour 2. Control over whether to engage in the behaviour (autonomy) 3. Can control how body responds to Covid-19 (control) 4. Perceived behavioural control over other people’s behaviour 5. Perceived behavioural control over other responsibilities such as work and childcare 6. Covid-19 can be cured / controlled 7. Response efficacy (behaviour will avert the threat) / Belief about effectiveness of the behaviour | Dixon, Jorgensen, Norman, Schuz, Lewis  Dixon  Eraso (a), Eraso (b), Hills  Norman, Schuz  Dixon  Eraso (b), Hills  Eraso (a), Eraso (b), Hills  Dixon  Dixon, Lewis, Fujii |
| 5. Optimism (n=0 constructs; n=0 studies) | - | - |
| 6. Beliefs about consequences  (n=35 constructs; n=16 studies) | 1. Risk perception (aggregate of risk to self, risk to others) 2. Risk perception (6 items aggregated - worried personally about Covid-19, how likely will be affected and catch Covid-19, how likely family and friends affected by Covid-19, will Covid -19 affect many people, will I get sick with Covid-19, getting Covid-19 is serious)   Consequences for self (n=21)   1. Perceived severity if catch Covid-19 2. Symptoms of Covid-19 will last a long time (consequences) 3. Consequences of Covid-19 for the individual (consequences) 4. Illness attitude (fears, attitudes, and beliefs associated with health) 5. Getting Covid-19 would make you anxious (emotion) 6. Affected by Covid-19 rules (mental health) 7. Affected by Covid-19 rules (physical health) 8. Affected by Covid-19 rules (economically) 9. Perceived costs of following govt advice: lose touch with friends and relatives 10. Perceived costs of following govt advice: get in trouble with police 11. Perceived costs of following govt advice: negative impact on finances 12. Perceived costs of following govt advice: won’t be able to carry out religious activities 13. Current lockdown is increasing conflict with people live with 14. Enjoying spending more time at home due to lockdown 15. Because of virus, feeling a sense of community with others in the neighbourhood 16. Experiential attitude (pleasant/unpleasant) 17. Instrumental attitude (harmful/beneficial) 18. Affective attitudes (behaviours pleasant /unpleasant) 19. Cognitive attitudes (behaviours harmful / beneficial) 20. Concern about personal safety and of close others^1^ 21. Concern about health impact of Covid-19 on self and family^1^ 22. Perceived severity of impact on self and family if catch Covid-19^1^   Consequences for family and friends (n=4)   1. Concern about personal safety and of close others^1^ 2. Perceived severity of impact on self and family if catch Covid-19^1^ 3. Concern about health impact of Covid-19 on self and family^1^ 4. Worry about consequences for self, family, friends^1^   Consequences for society (n=6)   1. Concern about health impact of Covid-19 on other members of society 2. Concern about effect of Covid-19 on NHS & health services 3. Perceived costs of following govt advice: if follow advice will protect the NHS 4. Concern about effect of Covid-19 on economy and infrastructure 5. Perceived costs of following govt advice: if follow advice will save lives 6. Utility perception - positive / negative consequences for you, people around you and society in general   Susceptibility   1. Perceived susceptibility of getting infected with Covid-19 2. If leave home, could catch covid-19 (susceptibility)   Transmission   1. If leave home could pass Covid-19 on to other people (transmission) 2. Perceived ease of transmission | Lewis  Schneider  Bowman, Dixon, Fujii, Smith (a)  Dixon  Dixon  Bacon  Dixon  Smith (a), Margraf  Smith (a), Margraf  Margraf  Smith (a)  Smith (a)  Smith (a)  Smith (a)  Smith (a)  Smith (a)  Smith (a)  Norman  Norman  Schuz  Schuz  Bacon  Jorgensen  Raihani  Bacon  Smith (a)  Raihani  Jorgensen  Raihani  Bacon  Smith (a)  Bacon  Smith (a)  Juanchich  Dixon, Eraso (a), Eraso (b), Fujii, Hills, Norman, Schuz  Smith (a)  Smith (a)  Bowman |
| 7. Reinforcement (n=0 constructs; n=0 studies) | - | - |
| 8. Intentions  (n=1 construct; n=5 studies) | 1. Intentions | Dixon, Eraso (a), Hills, Norman, Schuz |
| 9. Goals  (n=2 constructs; n=1 study) | 1. COM-B reflective motivation 2. COM-B automatic motivation | Armitage  Armitage |
| 10. Memory, attention and decision processes  (n=2 constructs; n=2 studies) | 1. Rational thinking style 2. Cognitive reflection (to inhibit intuitive thinking and adapt analytical thinking) | Swami  Juanchich |
| 11. Environmental context and resources  (n=8 constructs; n= 6 studies) | 1. Access to essentials 2. Financial support 3. COM-B physical opportunity (e.g. financial means) 4. Time use – remote working 5. Time use – home chores 6. Time use – arts and crafts 7. Time use – leisure 8. Time use – spending time outdoors | Wright/Steptoe  Eraso (a), Eraso (b), Hills  Armitage, Lewis  Wright (b)  Wright (b)  Wright (b)  Wright (b)  Wright (b) |
| 12. Social influences  (n=41 constructs; n=17 studies) | 1. COM-B social opportunity (interpersonal influences, cultural norms)   Engagement with other people   1. Face-to-face isolation (contact for 15mins) 2. Phone isolation (contact by phone or video) 3. Loneliness 4. Community group engagement   Norms   1. Normative pressure family 2. Normative pressure friends 3. Normative pressure neighbours 4. Friends and family disapprove if don’t follow rules 5. Injunctive norms (others approve) 6. Descriptive norms (others engage in behaviour) 7. Behavioural norms (other engage in behaviour) 8. Social norms (others engage in behaviour)   Trust   1. Trust in government 2. Trust in science 3. Social trust / interpersonal trust 4. Confidence in government 5. Confidence in health system   Beliefs about sources of information   1. Communication by govt and authorities credible and honest 2. Communication by govt and authorities guided by the interests of the people 3. Reliability of information about Covid19 sources: public institutions e.g. govt 4. Reliability of information about Covid-19 sources: social network services 5. Reliability of information about Covid-19 sources: family and friends 6. Reliability of information about Covid-19 sources: specialists - doctors and nurses 7. Reliability of information about Covid-19 sources: online news 8. Reliability of information about Covid-19 sources: radio 9. Reliability of information about Covid-19 sources: TV 10. Reliability of information about Covid-19 sources: information leaflet given at workplace/ school   Beliefs about support   1. Feel by national govt and authorities supported 2. Feel by national govt and authorities well informed 3. Feel by national govt and authorities taken seriously 4. Feel by national govt and authorities left alone 5. Support to do the behaviour 6. Social support – special person 7. Social support – family 8. Social support – friends 9. Social support – community 10. If helped someone outside of own household 11. Caring for a friend or relative 12. If received help from someone outside of own household 13. Support to self-isolate | Armitage  Wright (b)  Wright (b)  Wright (b)  Wright (b)  Eraos (a), Eraso (b), Hills  Eraso (b), Hills  Smith (a)  Norman, Smith (a)  Norman, Schuz  Dixon  Lewis  Smith (a)  Eraso (a), Eraso (b), Hills, Jorgensen, Juanchich  Maher  Jorgensen, Woelfert  Wright (b)  Wright(b)  Margraf  Margraf  Schiina  Schiina  Schiina  Schiina  Schiina  Schiina  Schiina  Schiina  Margraf  Margraf  Margraf  Margraf  Wright (b)  Wright (b)  Eraso (a), Eraso (b), Hills  Eraso (a), Eraso (b), Hills  Eraso (a), Eraso (b), Hills  Wright (b)  Smith (a)  Smith (a)  Lewis |
| 13. Emotion  (n=7 constructs; n=4 studies) | 1. Worry about getting Covid-19 (emotion) 2. Worry about Covid-19 3. Life worthwhile 4. Sleep quality 5. Number of life stressors 6. Life satisfaction (happiness) 7. Regional-level happiness | Dixon  Smith (a)  Wright (b)  Wright (b)  Wright (b)  Krekel, Wright (b)  Krekel |
| 14. Behavioural regulation  (n=2 constructs; n=2 studies) | 1. Information-seeking about Covid-19 2. Made plan so could do the behaviour | Wright (b)  Lewis |
| 15 Personality^2^  (n= 6 constructs; n=2 studies) | 1. Personality 2. Big 5 personality traits (optimism, neuroticism, extraversion, openness, conscientiousness) 3. risk-taking 4. locus of control 5. resilience 6. cognitive and emotional empathy | Bacon  Wright (a)  Wright (a)  Wright (a)  Wright (a)  Wright (a) |

^1.^ The construct was about consequences for self and for family and friends. Hence, there are 38 constructs listed but 3 are double-counted and so that total number of constructs is in fact 35.

^2^ Not a TDF but added

# Table S6: Psychological factors and theoretical domains associated with behaviour /intention

| **Author** | **Behaviour category** | **Behaviours** | **Psychosocial variables associated with higher behaviour adherence / intention** | **Psychosocial variables investigated and not found to be associated with behaviour / intention** |
| --- | --- | --- | --- | --- |
| Armitage | Adherence to government instructions | Adherence to government instructions | 1. People with greater physical capability, 2. psychological capability, 3. physical opportunity, 4. social opportunity, 5. reflective motivation & 6. automatic motivation | - |
| Atchison | - | - | - | - |
| Bacon | *Self-isolating*^1^ | *Self-isolating^1^* | *People with higher Fight-Freeze Flight System score, lower illness attitude, lower behavioural inhibition system^1^*  *(Concern about effect of Covid-19 on personal safety and concern about effect on NHS were correlated with self-isolation in bivariate analyses but not included in the multiple regression)* | *(Concern about effect of Covid-19 on economy was not correlated with self-isolation in bivariate analysis)* |
| Bowman | Social gatherings, and leaving the house  Contact with other people | (1) General: Avoiding crowds, social events, going out  (2) Contact: avoiding contacting individuals who had a fever or respiratory symptoms and/or had been in affected areas of UK  (3) Avoiding going to work | 1. Perceived severity, 2. perceived transmission of covid-19 easy (transmission) 3. Perceived severity, 4. perceived transmission of Covid-19 easy (transmission) 5. Perceived severity | 1. Perceived transmission of Covid-19 easy |
| Dixon | Physical distance  Face covering  Hand hygiene | 2 metre distancing when outside the home  Face covering when in shop, on public transport  Washed hand as soon as got home, before eating snacks, washed hands for 20 seconds, washed hands with soap and water | 1. People with higher self-efficacy, 2. intention, 3. being ill with Covid-19 will last a time (timeline), 4. Covid-19 is a recurrent disease (timeline), 5. other people not keeping distance is a cause of Covid-19 (cause), 6. anxiousness (emotional consequence), 7. perceived severity, 8. response efficacy, 9. response self-efficacy 10. People with higher self-efficacy (response self-efficacy), 11. intention, 12. behavioural norm, 13. Covid-19 is a recurrent disease (timeline), 14. consequences for individual will be high (consequences), 15. not washing hands is a cause of Covid-19 (cause), 16. not wearing face covering is a cause of Covid-19 (cause), 17. other people not keeping distance is a cause of Covid-19 (cause), 18. worry (emotion), 19. perceived severity, 20. response efficacy, 21. response self-efficacy, 22. People with higher self-efficacy, 23. intention, 24. being ill with Covid-19 will last a time (timeline), 25. Covid-19 is a recurrent disease (timeline), 26. not wearing face covering is a cause of Covid-19 (cause), 27. other people not keeping distance is a cause of Covid-19 (cause), 28. perceived severity, 29. response efficacy, 30. response self-efficacy | 1. Behavioural norm (descriptive norm), 2. consequences of Covid-19 for individual, 3. perceived risk (susceptibility) 4. Covid-19 can be cured (control) 5. Can control how body responds to Covid-19 (control) 6. Symptoms of Covid-19 easy to recognise (identity) 7. Perceived risk (susceptibility) 8. Covid-19 can be cured (control) 9. Can control how body responds to Covid-19 (control) 10. Behavioural norm (descriptive norm), 11. consequences of Covid-19 for individual, 12. emotion, 13. perceived risk (susceptibility) 14. Covid-19 can be cured (control) 15. Can control how body responds to Covid-19 (control) |
| Dowthwaite | - | - | - | - |
| Eraso^1^ (b) | *Self-isolating^1^* | *Self-isolating^1^* | *(1) OWN SYMPTOMS:*   1. *People with higher perceived behavioural control over leaving house,* 2. *higher perceived behavioural control over other responsibilities e.g. work and childcare(n=2)*   *(2) CO-HABITANT SYMPTOMS:*   1. *People with higher perceived susceptibility,* 2. *greater knowledge of Covid-19 rules,* 3. *higher perceived behavioural control over leaving house,* 4. *lower community support (n=4)* | 1. *Perceived susceptibility,* 2. *knowledge about Covid-19 rules,* 3. *social responsibility,* 4. *self-interest,* 5. *political party voted for,* 6. *financial support,* 7. *normative pressure of family,* 8. *trust in government,* 9. *support from special person,* 10. *support from family,* 11. *support from friends,* 12. *support from community, (n=12)* 13. *Social responsibility,* 14. *self-interest,* 15. *political party voted for,* 16. *financial support,* 17. *normative pressure of family,* 18. *trust in government,* 19. *support from special person,* 20. *support from family,* 21. *support from friends,* 22. *higher perceived behavioural control over other responsibilities e.g. work and childcare (n=10)* |
| Eraso^1^ (a) | Leaving the house  Leaving the house | (1) Intentional to go out to meet up with family, go out to meet up with friends and go out for unpermitted reasons  (2) Went out to meet up with family, went out to meet up with friends, went out for unpermitted reasons, went out for permitted reasons but unable to keep 2 metre physical distance (parks and public spaces for exercise, went out for groceries, went out for medication) | 1. People with higher trust in government, 2. greater knowledge about Covid-19 rules, 3. higher sense of social responsibility, 4. higher perceived intention, 5. higher perceived control over other’s distancing behaviour, 6. higher feeling of normative pressure from friends to socially distance, 7. lower feeling of normative pressure from neighbours to socially distance, 8. lower support from friends (n=8) 9. Higher perceived intention, 10. control over others social distancing, 11. control over leaving the house, 12. lower perceived support from friends, 13. lower perceived normative pressure from neighbours (n=5) | 1. Perceived susceptibility, 2. political party voted for, 3. self-interest, 4. personal control over leaving house, 5. behavioural control over other responsibilities e.g. work and childcare, 6. normative pressure of family, 7. financial support, 8. support from special person, 9. support from family, 10. support from community,   (n=10)   1. Perceived susceptibility, 2. social responsibility, 3. self-interest, 4. political party voted for, 5. trust in government, 6. knowledge about Covid-1 rules, 7. behavioural control over other responsibilities e.g. work and childcare, 8. normative pressure of family, 9. normative pressure of friends, 10. support from special person, 11. support from family, 12. support from community, 13. financial support (n=13) |
| Fujii | Social gatherings  Face covering  Hand hygiene | Social gatherings of more than 20 people  Face covering  Use hand sanitiser, wash hands with soap and water | 1. Perceived effectiveness   1. Perceived effectiveness, 2. Perceived susceptibility   1. Perceived effectiveness | 1. Perceived severity, 2. perceived susceptibility   1. Perceived severity   1. Perceived severity, 2. perceived susceptibility |
| Galasso | - | - | - | - |
| Hills^1^ | Leaving the house  Leaving the house | (1) Intentional to go out to meet up with family, go out to meet up with friends and go out for unpermitted reasons  (2) Went out to meet up with family, went out to meet up with friends, went out for unpermitted reasons, went out for permitted reasons but unable to keep 2 metre physical distance (parks and public spaces for exercise, went out for groceries, went out for medication) | 1. People who did not vote for the government, 2. higher intention, 3. higher perception of control over other people’s distancing, 4. lower perceived normative pressure from neighbours, 5. lower support from friends (n=5) 6. People with higher perception of control over other people’s distancing 7. people with lower perception of control over their other responsibilities such as work and childcare (n=2) | 1. Perceived susceptibility, 2. social responsibility, 3. self-interest, 4. trust in government, 5. knowledge about Covid-19, 6. personal control over leaving the house, 7. behavioural control over other responsibilities e.g. work and childcare, 8. normative pressure of family, 9. normative pressure of friends, 10. support from special person, 11. support from family, 12. support from community, 13. financial support (n=13) 14. Intention to social distance, 15. perceived susceptibility, 16. social responsibility, 17. self-interest, 18. political party voted for, 19. trust in government, 20. knowledge about Covid-19, 21. personal control over leaving the house, 22. normative pressure of family, 23. normative pressure of friends, 24. normative pressure of neighbours, 25. support from special person, 26. support from family, 27. support from friends, 28. support from community, 29. financial support (n=16) |
| Jain | - | - | - | - |
| Jorgensen | Physical distance, contact with other people, social gatherings and travel  Hand hygiene, other hygiene behaviours and cleaning | Contact with elderly and chronically ill people, hugging or kissing outside of close family members, shaking someone’s hand and meeting in room with ≥10 people and use of public transport  Washed hands with sanitiser, coughing into one’s sleeve and cleaning | 1. People with higher self-efficacy, 2. higher worry about consequences 3. higher institutional trust 4. people with lower interpersonal trust (n=4) 5. People with higher self-efficacy, 6. higher worry about consequences 7. higher institutional trust 8. people with lower interpersonal trust (n=4) | - |
| Keyworth | - | - | - | - |
| Lawson | - | - | - | - |
| MacIntyre | - | - | - | - |
| Maher | Physical distance and hand hygiene | Physical distancing and clean hands | People with lower science scepticism | 1. Views on Brexit, 2. political orientation |
| Margraf | Adherence to government instructions | Adherence to government instructions | 1. Affected by Covid-19 (physical health) 2. Affected by Covid-19 (mental health) (n=2) | 1. Communication clear and understandable from govt and authorities 2. Communication from govt credible and honest 3. Communication by govt guided by interests of people 4. Feel well supported by govt and authorities 5. Feel well informed by govt and authorities 6. Feel taken seriously by govt and authorities 7. Feel left alone by govt and authorities 8. Affected by Covid-19 (economically)   (n=8) |
| Norman | Physical distance  Physical distance  Leaving the house  Leaving the house  Hand hygiene | (1) 2 metre distancing when inside a shop  (2) Physical distance - 2 metre distancing when outside the home  (3) Meet family or friends  (4) Going out for food shopping, exercise, medical needs or travelling to work  (5) Washed hands as soon as got home | 1. Experiential attitudes 2. people with higher capacity (self-efficacy) (n=2) 3. People with higher perceived susceptibility, 4. higher capacity (self-efficacy) 5. higher intention (n=3) 6. People with higher intention 7. people with higher capacity (self-efficacy) (n=2) 8. People with higher instrumental attitudes (if doing behaviour would be beneficial or harmful) 9. people with higher capacity (self-efficacy) (n=2) 10. People with higher experiential attitudes, 11. higher capacity (self-efficacy), 12. higher control over whether to do the behaviour (autonomy) 13. higher intention (n=4) | 1. People with higher perceived susceptibility, 2. instrumental attitudes, 3. control over whether to do the behaviour (autonomy), 4. intention, 5. injunctive norm, 6. descriptive norm (n=6) 7. instrumental attitudes, 8. experiential attitudes, 9. control over whether to do the behaviour (autonomy), 10. injunctive norm, 11. descriptive norm (n=5) 12. People with higher perceived susceptibility, 13. instrumental attitudes, 14. experiential attitudes, 15. control over whether to do the behaviour (autonomy) 16. injunctive norm, 17. descriptive norm (n=6) 18. People with higher perceived susceptibility, 19. control over whether to do the behaviour (autonomy), 20. intention, 21. injunctive norm, 22. descriptive norm, 23. experiential attitudes (n=6) 24. People with higher 25. perceived susceptibility, 26. injunctive norm, 27. descriptive norm, 28. instrumental attitudes (n=4) |
| Perrotta | - | - | - | - |
| Schneider | Leaving the house  Leaving the house  Leaving the house  Travel  Face covering  Hand hygiene  Hand hygiene  Other hygiene behaviours | Going out for groceries  Cooking at home  Purchasing extra supplies  Public transport  Face covering  Washing hands more often  Used hand sanitiser  Touching face | People with higher risk perception  People with higher risk perception  People with higher risk perception  People with higher risk perception  People with higher risk perception  People with higher risk perception  People with higher risk perception  People with higher risk perception | - |
| Schuz | Physical distance, leaving the house, face covering, hand hygiene | 2 metre distance inside shop, 2 metre distance when outside home, visiting family and friends, limiting to one session of exercise a day, leave home to shop for food, go out for food shopping, exercise, medical needs or travelling to work, face covering when away from home, washing hands as soon as got home | 1. People with higher intention, 2. higher affective attitude, 3. higher descriptive norms, 4. higher capability (self-efficacy), 5. higher autonomy, 6. higher perceived susceptibility 7. lower previous past behaviour (n=7) | 1. Cognitive attitude, 2. injunctive norms (n=2) |
| Shiina | Face covering | Face covering | People who perceive that: i) social networks, ii) friends and neighbours are reliable and credible sources of Covid-19 information and people who do not perceive that i) specialists (doctors and nurses), and ii) official announcements by government are reliable and credible sources of Covid-19 information (n=4) | Reliability of information from:   1. online networks, 2. radio, 3. TV, 4. workplaces (n=4) |
| Smith (a) | Leaving the house | Left home to go to the shops for groceries, toiletries or medicine; to go to the shops for other items; for exercise; for a medical purpose excluding going to the shops/pharmacy for medicine; to go to work; to help someone else; and to meet friends or family who they did not live with | GROUP 1 IF HAVE SYMPTOMS IN HOUSEHOLD:   1. people who do not think that the lockdown had made their mental health worse, 2. people who do not feel a greater sense of community with their neighbourhood due to Covid-19, 3. people who have increased worry about Covid-19, 4. people who have received help from someone outside of their household because of Covid-19, 5. people with higher perceived likelihood of catching Covid-19 (n=5)   GROUP 2 IF NO SYMPTOMS IN HOUSEHOLD:   1. people who were not helping someone outside of their household due to Covid-19, 2. people who were receiving help from someone outside of their household, 3. people who did not think that they would lose touch with friends and relatives if they followed government advice, 4. people who enjoyed spending more time at home during the lockdown, 5. people with higher perceived severity of Covid-19 (severity) 6. people with higher perceived likelihood of spreading Covid-19 (transmission) 7. people with higher perceived legal consequences of not following government advice, 8. people with higher perceived social pressure from friends and family to follow government measures, 9. people not knowing or being unsure about government measures, 10. people who did not think that following government advice would negatively impact them financially, 11. people with higher perceived social norms, 12. people with lower perceived impact of lockdown on physical health, 13. people with higher worry about Covid-19, 14. people with higher perceived likelihood of catching Covid-19 (susceptibility) (n=14) | 1. Understanding of government measures, 2. Perceived severity of impact on family well-being if catch Covid-19, 3. If leave home could pass Covid-19 on to other people (transmission), 4. People who perceived Covid-19 will be severe if catch it, 5. Affected by Covid-19 (physical health), 6. Perceived costs of following govt advice: lose touch with friends and relatives, 7. Perceived costs of following govt advice: get in trouble with police, 8. Perceived costs of following govt advice: if follow advice will save lives, 9. Perceived costs of following govt advice: if follow advice will protect the NHS, 10. Perceived costs of following govt advice: negative impact on finances, 11. Perceived costs of following govt advice: won’t be able to carry out religious activities, 12. Current lockdown is increasing conflict with people live with, 13. Enjoying spending more time at home due to lockdown, 14. Social norms, 15. Friends and family disapprove if don’t follow rules, 16. If helped someone outside of own household (n=16) 17. Affected by Covid-19 (mental health),      1. Perceived severity of impact on family if catch Covid-19, 2. Perceived costs of following govt advice: if follow advice will save lives, 3. Perceived costs of following govt advice: if follow advice will protect the NHS, 4. Perceived costs of following govt advice: won’t be able to carry out religious activities, 5. Current lockdown is increasing conflict with people live with, 6. Because of virus, feeling a sense of community with others in the neighbourhood, (n=7) |
| Smith (b) | - | - | - | - |
| Smith (c) | - | - | - | - |
| Swami | Physical distance, leaving house, and hand hygiene | 2 metre distance outside home, visiting family and friends, leaving house for food shopping, exercise, medical needs or travelling to work, washed hand as soon as got home | 1. Rational thinking style 2. Lower Covid-19 conspiracy theories |  |
| Woelfert | Physical distance, contact with other people, social gatherings | Keep safe distance from others, avoid people who cough and sneeze, social events, | Lower social trust in others |  |
| Wright (a) | Adherence to government instructions | Adherence to government instructions | 1. People with lower risk-taking, 2. people with lower external locus of control, 3. people with higher optimism, 4. people with higher resilience, 5. people with higher neuroticism, 6. people with higher agreeableness, 7. people with lower extraversion, 8. people with higher openness, 9. people with higher conscientiousness, 10. people with higher cognitive and emotional empathy (n=10) |  |
| Wright (b) | Adherence to government instructions | Adherence to government instructions | 1. higher confidence in government, 2. higher confidence in the health service, 3. people with greater access to essentials, 4. People with perceived fewer stressors, 5. people with higher happiness, 6. people with higher perceived sense that life’s activities were worthwhile, 7. people with better sleep quality, 8. People with lower loneliness, 9. people with higher face-to-face contact 10. people with higher telephone contact, 11. People with higher levels of knowledge and information seeking relating to COVID-19 12. People not working outside the house 13. People not caring for a friend or relative 14. People who were engaging with a community group 15. People who were spending time on arts and craft, 16. People who were spending time on leisure, 17. People who were spending time doing household chores, 18. People who were working remotely | 1. Time spent outdoors, |
| Juanchich | Leaving the house  *Testing^1^*  Face covering  Other hygiene behaviours  *Testing* | (1) Stockpiling  *(2) Intention to get tested^1^*  (3) Face masks  (4) Gloves  *Intention to get tested*    *Intention to install Test & Trace App^1^* | None   1. *People with lower Covid-19 conspiracy beliefs* 2. *People with higher trust in govt* 3. People with higher Covid-19 conspiracy beliefs 4. People who with higher belief that behaviour would have positive consequences (utility perception) 5. People with higher Covid-19 conspiracy beliefs 6. People who with higher belief that behaviour would have positive consequences (utility perception) 7. *People with lower Covid-19 conspiracy beliefs* 8. *People who with higher belief that behaviour would have positive consequences (utility perception)*   None | 1. general conspiracy beliefs 2. Covid-19 conspiracy beliefs 3. Trust in govt (n=3) 4. *Cognitive reflection (analytic thinking) (n=1)*   1. Political ideology   1. Political ideology   *1. Political ideology*  *1. Covid-19 conspiracy beliefs*  *2. behaviour would have positive consequences (utility perception)* |
|  | *Covid-19 testing^1^*  *Covid-19 testing^1^* | *Getting tested^1^* | *People with higher trust in government*  *lower general conspiracy beliefs,*  *people with higher perceived utility of the behaviour^1^*  *People with lower coronavirus conspiracy beliefs, people with higher* *perceived utility of the behaviour^1^* |  |
| Krekal | Leaving the house  *Self-isolate*  Leaving the house, social gatherings, hand hygiene, traveling, face covering, other hygiene behaviours, avoiding contact with other people, cleaning | (1) Staying at home  *(2) willing to isolate*  (3) Aggregate of behaviours | Regional level happiness  People with higher life satisfaction (happiness)  People with higher life satisfaction (happiness) |  |
| Raihani | Leaving the house, contact with other people, social gatherings, hand hygiene, other hygiene behaviours, travel | Staying at home, working from home, working events, stockpiling, contact with elderly and chronically ill people, social events, travel within UK, travel abroad, washing hands more often, touching face | 1. People who are more concerned about health impacts of Covid-19 on self and on family 2. People who are more concerned about health impacts of Covid-19 on other members of society (n=2) | 1. Political ideology |
| Lewis | *Self-isolating^1^* | *Self-isolating^1^* | 1. Knowledge - people who believe that they know how to self-isolate (psychological capability) 2. Beliefs about effectiveness of behaviour 3. Made plans so could self-isolate (n=3) | 1. Physical opportunity (financial means to self-isolate) 2. Risk perception, 3. Self-efficacy 4. Support to self-isolate 5. Social norms 6. Past self-isolation behaviour (n=6) |
| Gould | - | - | - | - |

^1.^ The number 1 and italics denotes that the independent variable is behaviour intention
